# Pan-cancer quantitation of epithelial-mesenchymal transition dynamics using parallel reaction monitoring-based targeted proteomics approach

**DOI:** 10.1186/s12967-021-03227-0

**Published:** 2022-02-11

**Authors:** Ankit P. Jain, Janani Sambath, Gajanan Sathe, Irene A. George, Akhilesh Pandey, Erik W. Thompson, Prashant Kumar

**Affiliations:** 1 Institute of Bioinformatics, International Technology Park, Bangalore, 560066 Karnataka India; 2 Manipal Academy of Higher Education (MAHE), Manipal, 576104 India; 3 Center for Molecular Medicine, National Institute of Mental Health and Neurosciences (NIMHANS), Bangalore, 560029 India; 4 Department of Laboratory Medicine and Pathology, Centre for Individualized Medicine, Mayo Clinic, Rochester, MN 55905 USA; 5 Institute of Health and Biomedical Innovation and School of Biomedical Sciences, Queensland University of Technology, Brisbane, QLD 4059 Australia; 6 School-Biomedical Sciences, Translational Research Institute, Woolloongabba, QLD 4102 Australia; 7 Somaiya Institute of Research and Consultancy (SIRAC), Somaiya Vidyavihar University (SVU), Vidyavihar, Mumbai, 400077 Maharashtra India

**Keywords:** Epithelial-mesenchymal transition, Pan-cancer targeted proteomics, Parallel reaction monitoring, Mass spectrometry

## Abstract

Epithelial–mesenchymal transition (EMT) is a dynamic and complex cellular process that is known to be hijacked by cancer cells to facilitate invasion, metastasis and therapeutic resistance. Several quantitative measures to assess the interplay between EMT and cancer progression are available, based on large scale genome and transcriptome data. However, these large scale multi-omics studies have repeatedly illustrated a lack of correlation in mRNA and protein abundances that may be influenced by diverse post-translational regulation. Hence, it is imperative to understand how changes in the EMT proteome are associated with the process of oncogenic transformation. To this effect, we developed a parallel reaction monitoring-based targeted proteomics method for quantifying abundances of EMT-associated proteins across cancer cell lines. Our study revealed that quantitative measurement of EMT proteome which enabled a more accurate assessment than transcriptomics data and revealed specific discrepancies against a backdrop of generally strong concordance between proteomic and transcriptomic data. We further demonstrated that changes in our EMT proteome panel might play a role in tumor transformation across cancer types. In future, this EMT panel assay has the potential to be used for clinical samples to guide treatment choices and to congregate functional information for the development and advancing novel therapeutics.

**Supplementary Information:**

The online version contains supplementary material available at 10.1186/s12967-021-03227-0.

## Introduction

Epithelial-mesenchymal transition (EMT) is a cellular process that allows transdifferentiation of cells with a polarized epithelial phenotype to gain mesenchymal characteristics. It is a highly coordinated process that is regulated at genetic, epigenetic and protein levels by different regulators [1–3]. Epithelial cells show inherent plasticity that covers a range of changes in cellular behaviour and differentiation characteristics with epithelial integrity at one end and a complete mesenchymal transition on the other end [4]. Epithelial cells may simultaneously express varying levels of both epithelial and mesenchymal characteristics depending on the tissue and signalling context, exhibiting a partial EMT phenotype and exist in an intermediate cell state [5, 6]. In our previous study, we employed an EMT scoring method to compute the generic EMT scores from transcriptome datasets. Our study revealed intermediate EMT phenotype in circulating tumor cells (CTCs) across cancers [7]. In our recent work, we have identified 5 categories of CTCs ranging from E (exclusively) to E > M, E = M, M > E and M (exclusively) suggesting dynamic changes in epithelial and mesenchymal composition supported by other published work in the field [8, 9]. Thus, it is of paramount importance to understand the EMT spectrum in cancers.

Several signalling cascades and downstream transcriptional regulators such as SNAIL, TWIST and ZEB are known to be associated with EMT [10, 11]. Advanced technology and cell biology-based approaches have immensely improved our understanding of molecular mechanisms of EMT over the past decades [12]. Nevertheless, such approaches are usually restricted in the number of targets that can be simultaneously monitored. High throughput technologies such as transcriptomics dominated the investigation of EMT models in numerous studies [13–16]. However, mRNA levels estimation may not correlate with protein expression due to a range of post-translational regulations [17–19]. Thus, investigating protein expression changes that are associated with changes in cellular phenotype would provide us an exceptional understanding of mechanisms and functionalities related to EMT.

Mass spectrometry and antibody arrays have been used to assess protein expression dynamics. Although mass spectrometry-based proteomics studies offered us to estimate quantative differential expression of many proteins associated with EMT process under different biological contexts [20, 21], these platforms are limited in their range and sensitivity as well as their ability to consistently detect the absolute protein quantification [22–24]. Thus, establishing a robust method to effectively monitor proteomic changes associated with EMT is essential for further understanding of the complex regulation involved in EMT.

In previous studies most of the approaches employed either the transcriptomics analysis or the mathematical modeling and were focused on classifying the dynamic state of the cellular phenotypes [25, 26]. However in this study we intended to identify the global changes at the protein levels using parallel reaction monitoring (PRM)-based targeted proteomics assay as a tool for the absolute quantification of the proteins involved in these dynamic changes. A mass spectrometry-based, targeted proteomics strategy would be relatively fast and highly reproducible [27, 28]. This method allows quantification down to attomole range in a straightforward way without any prior enrichment or fractionation approaches [27, 29]. We observed the relative expression of the established panel of EMT-related proteins that distinguishes between epithelial and mesenchymal cellular phenotypes. Most of the cell lines showed synergism between protein expression and gene expression. However, some cell lines exhibited distinguished protein expression compared to gene expression. Further, our study also showed that this method can be applied to tumor tissues as well for the characterization of tumor phenotype.

## Results

### Expression of epithelial and mesenchymal genes across pan-cancer cell lines

**Fig. 1:**
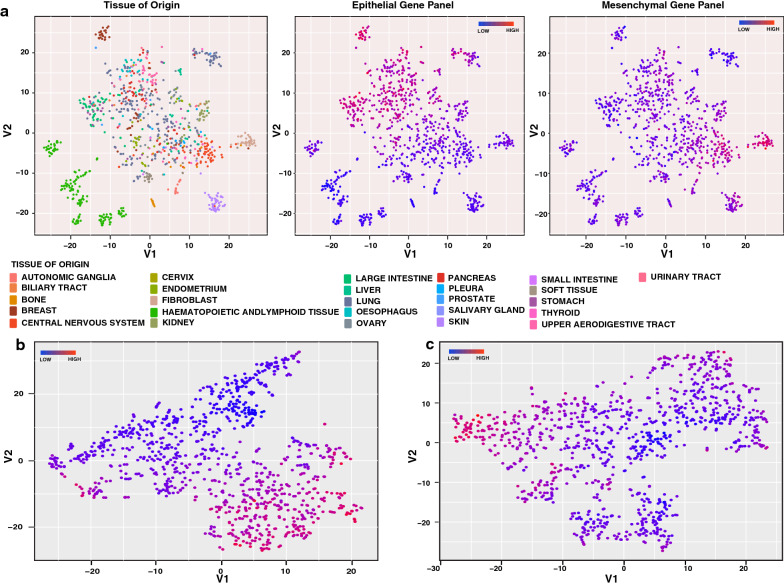
Pan-cancer cell lines organized based on transcriptomics data from CCLE database. **a** t-SNE plots of cancer cell lines based on global transcriptome data. Each point represents a cell line and is coloured by the tissue of origin (1), expression of epithelial genes (2), or expression of mesenchymal genes (3). **b** t-SNE plots of cancer cell lines based on expression of epithelial genes. **c** t-SNE plots of cancer cell lines based on expression of mesenchymal genes

EMT is known to play an important role in oncogenic transformation. To examine EMT in the context of the oncogenic transformation of different organ types, we analyzed transcriptome data across different cancer types from Cancer Cell Line Encyclopedia (CCLE) using t-Distributed Stochastic Neighbor Embedding (t-SNE) method [30, 31]. We observed that cancer cell lines generally clustered primarily based on tissue of origin (Fig. 1a, left panel). Tight clusters were observed for renal, breast, fibroblast, skin, and hematopoietic cell lines, whereas lung cancer cell lines showed a scattered cluster. We curated a list of 37 genes from the literature belonging to multiple cellular processes associated with EMT phenotype (Additional file 1: Fig. S1). To test whether this panel of genes can distinguish epithelial and mesenchymal cell lines, we coloured the points on the t-SNE plot using the median z-score of epithelial and mesenchymal marker expression (Fig. 1a, middle, right panels). From the t-SNE analysis, we observed divergent organization of cells based on the expression of this curated set of epithelial and mesenchymal genes. Breast and gastric cancer cell line clusters showed high expression of epithelial markers, whereas cell lines from renal cancers and fibroblasts showed high expression of mesenchymal markers. Many cancers showed evidence of both. We also plotted t-SNE maps based on selected epithelial and mesenchymal gene expression values across these cell lines and coloured the points using median z-score. t-SNE analysis showed distinct clustering of cell lines based on the expression of the curated genes (Fig. 1b, c). These results indicate that the expression of EMT-related genes plays an essential role in governing the cellular plasticity across different cancer types. However, protein expression data of these EMT related genes across different cancer types are lacking.

### Development of PRM-assay panel for EMT-associated proteins

**Fig. 2:**
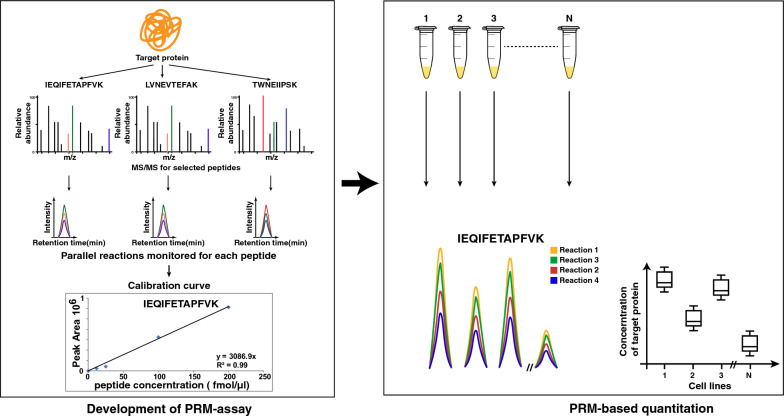
PRM-based quantitation of EMT associated proteins across a panel of cancel cell lines. Workflow illustrating the targeted proteomics-based evaluation of EMT-associated proteins in cancer cell lines. The time-scheduled Parallel reaction monitoring (PRM) assay was developed by monitoring more than 3 product ion transitions per peptide precursor. Reverse calibration using synthetic light and SIL peptides was generated to assess LOD, LOQ and linearity. SIL peptides were added to peptide digest from each cell line for normalization and quality assessment and quantitation

Compared to RNA, proteins are more closely related to cellular phenotype and hence it is important to quantify protein levels of EMT-related markers. Currently, most of the studies focus on quantation of RNA expression and there is no high throughput protein level data to monitor changes related to EMT. The overarching objective of this study was to develop a multiplex PRM method to quantify EMT-associated proteins across a panel of cancer cell lines including lung, head and neck squamous cell carcinoma, urinary bladder, gall bladder, gastric and ovarian cancer. A panel of 37 well established EMT-associated proteins were curated from previously published studies [25]. Selection of suitable peptides for PRM analysis is one of the most critical parameters for the development of a PRM-based proteomics assay. We followed the current standards in targeted proteomics for the selection of proteotypic peptides for PRM analysis [29, 32–35]. Following these selection criteria, we finalized 116 peptides corresponding to 37 proteins for the development of the PRM assay. The list is provided in Additional file 2: Table S1. For these selected peptides corresponding heavy amino acid labelled peptides (C-terminal 15 N and 13C-labeled arginine or lysine residues) were synthesized to generate a robust PRM method (Fig. 2).

The PRM assay was optimized for the detection of these stable isotope labelled (SIL) peptides in a complex mixture of the cell line peptides. Only peptides that were consistently detected were regarded as detectable targets and used for further analysis. Using the peptide retention time (RT), we generated a time-scheduled PRM method that analyzes 31 target proteins (96 endogenous peptides + 96 SIL peptides), allowing 10 min time windows for monitoring across cell lines. Performance of the assay was assessed by evaluating linearity, LOQ-, and LOD using a reverse calibration curve strategy. A set of 31 best peptides corresponding to 31 proteins were selected based on quality control criteria specified in the methods section, and consistent detection across all the cell lines with 3 or more PRM transitions per peptide for quantitation during data analysis using skyline software (Additional file 1: Fig. S2). The limit of detection for all the peptides was found to be in the range of 100 attomoles to 1 femtomoles. The lower limit of quantitation for most peptides was found to be in the range of 0.2–20 femtomoles (Additional file 1: Fig. S3, Additional file 3: Table S2).

### Comparison of transcriptomic and PRM-based proteomics profile for EMT-associated proteins

Data for all the 31 proteins were analyzed across 18 cell lines from 8 tumor types in technical triplicates using Skyline software. Some proteins were below the detectable limit of 100 attomole in many cell lines due to low stoichiometry; hence further analysis was restricted to a total of 20 proteins (Additional file 1: Fig. S3a, Additional file 4: Table S3). Epithelial cell lines such as Cal27, FaDu and MCF7 showed high expression of epithelial proteins and low expression of mesenchymal proteins. Similarly, we observed that mesenchymal cell lines such as MDAMB231, UMUC3 and J82 showed low expression of epithelial proteins and high expression of mesenchymal proteins.

**Fig. 3:**
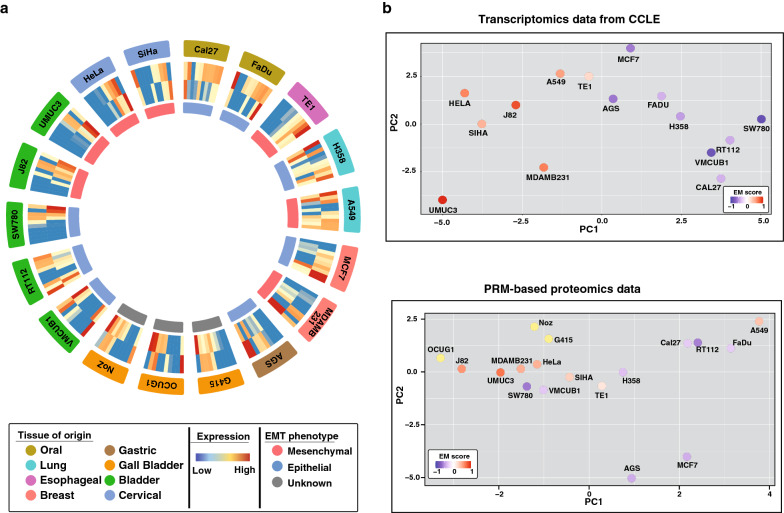
Transcriptome and proteome-based clustering of pan-cancer cell lines. **a** Expression of Epithelial and mesenchymal proteins across a panel of cancel cell lines. Layer from outside to inside: layer 1: tissue of origin, layer 2: expression of epithelial proteins (Clockwise-KRT18, KRT8, ANXA4, SDC1, EPCAM, CDH1, DSG3, MUC1, S100A14, SCNN1A), layer 3: expression of mesenchymal proteins (Clockwise-ITGA5, LAMC1, VIM, ITGB4, TUBA, LAMC2, FN, COL6A1, ITGB6, ZEB1), layer 4: EMT phenotype. **b** Principal component analysis of cell lines based on transcriptomics data from CCLE or PRM-based proteomics data coloured based on EMT score from Tan et al.

Further, we carried out Principal Component Analysis (PCA) using CCLE mRNA gene count data as well as PRM-based proteomic data for the EMT-related markers. PCA results showed consistent clustering of epithelial and mesenchymal cell lines in both the data sets (Fig. 3b). The epithelial and mesenchymal nature of these cell lines were inferred from the EMT score derived using transcriptomic EMT signatures of ovarian, breast, bladder, lung, colorectal and gastric cancers and the two-sample Kolmogorov–Smirnov-based method by Tan et al. [25]. Degree of EMT score ranges from − 1.0 to + 1.0 and cell lines with a positive EMT score exhibits a more mesenchymal phenotype, whereas a negative EMT score reflects a more epithelial phenotype. Proteomic analyses of EMT-related proteins also included three mesenchymal gall bladder cancer cell lines (viz*.* G-415, NoZ, and OCUG1) for which transcriptomics data is not available in the CCLE database. We observed clustering of these gall bladder cancer cell lines along with other cells showing mesenchymal phenotype in the proteomics data. This indicates that proteomics analysis can be useful in assigning the cellular phenotypes. However, certain outliers such as lung cancer cell line A549, and bladder cancer cell lines SW780 and VMCUB1 were also observed in the proteomics data, indicating proteomic heterogeneity.

Discordances between protein and transcript profile of cancer cell lines and tumors have been reported in multiple studies [36–38]. We compared the protein and mRNA expression of these cell lines using RPPA and transcriptomics data from CCLE database. We observed a low correlation score for the 3 outlier cell lines viz*.* A549, VMCUB1 and SW780, compared to epithelial cell lines such as RT112 and MCF7 and mesenchymal cell lines such as UMUC3 and MDAMB231 (Additional file 1: Fig. S4). Thus, understanding these exceptions in the context of EMT may be of importance and PRM-based proteomics assays can be a sensitive and versatile tool to assess the EMT proteome as this method permits an edge over the transcriptomics. In PRM-based proteomics assays we identify the absolute quantification of the proteins in each state (either epithelial or mesnchymal). However, the transcriptomics represent the relative changes of the expression of the gene in the cellular states.

### Comparative assessment of EMT by transcriptome and proteome analysis

**Fig. 4:**
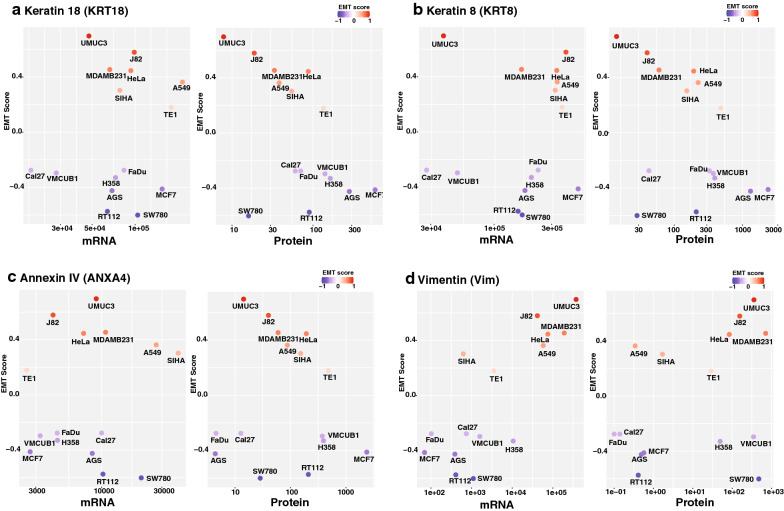
Expression of known epithelial and mesenchymal proteins across cell lines. a Expression of epithelial marker Keratin18 (KRT18) across cell lines vs EMT score (Epithelial = Blue to Mesenchymal = Red). **b** Expression of epithelial marker Keratin8 (KRT8) across cell lines vs EMT score (Epithelial = Blue to Mesenchymal = Red). **c** Expression of epithelial marker ANXA4 (Annexin IV) across cell lines vs EMT score (Epithelial = Blue to Mesenchymal = Red). **d** Expression of mesenchymal marker VIM (Vimentin) across cell lines vs EMT score (Epithelial = Blue to Mesenchymal = Red)

Multiple studies have reported the EMT signature across different cancer types using platforms such as genomics and transcriptomics [39, 40]. A benchmark study by Tan et al. computed the EMT score across cell lines and tumor samples based on transcriptomics data available in CCLE and TCGA [25]. We compared our PRM-based proteomics data with transcriptome-based EMT scores available for the cell lines that are common between the two studies. We observed higher protein levels of epithelial markers such as Keratin 8 and Keratin 18 in known epithelial cell lines such as MCF7, RT112 and AGS correlated with low EMT score (Fig. 4a, b). Similarly, we observed comparatively lower levels of these proteins in known mesenchymal cell lines such as MDAMB231, J82 and UMUC3 with higher EMT scores (Fig. 4a, b). On the contrary, we observed low expression of epithelial proteins Keratin 8, Keratin 18 and Annexin IV in SW780 cell line with low EMT score, whereas mRNA expression of these proteins were high (Fig. 4a–c).

Further, for the most part, high expression of mesenchymal marker vimentin (VIM) was observed in cell lines with high EMT score (Mesenchymal phenotype) and low expression was observed in cell lines with low EMT score (Epithelial phenotype) at both protein and transcript levels, concordant with their epithelial or mesenchymal phenotype (Fig. 4d). However, VMCUB1 and SW780 with low EMT scores showed a high amount of vimentin (VIM) at the protein level. Similarly, we observed low vimentin protein levels in the A549 cell line, which has a high EMT score. However, we observed no such discordant expression of vimentin for these cell lines at the mRNA level. This outlier protein expression pattern in some cell lines (viz. A549, SW780, and VMCUB1) could be one of the features related to the exceptional nature of these cell lines, as shown in Fig. 3b. However, additional studies are necessary to fully understand the cellular mechanisms that govern such contrasting expression patterns and their role in the cellular phenotype.

### Expression of EMT related proteins in tumor tissue samples from CPTAC database

**Fig. 5:**
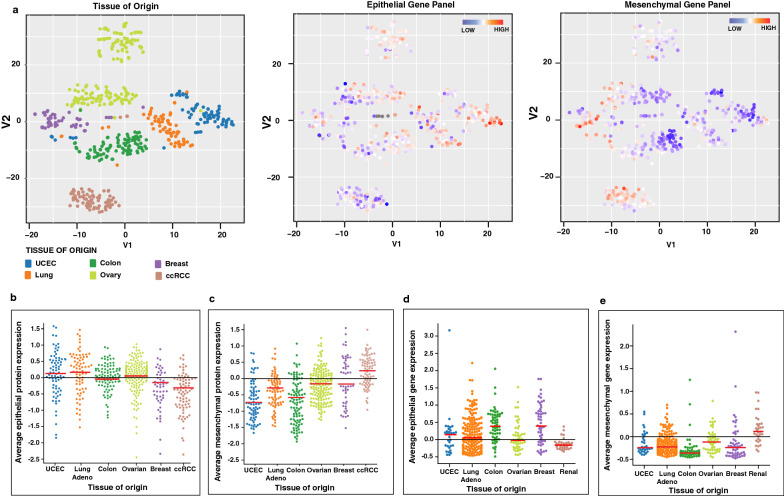
Pan-cancer tumor tissue samples organized by expression of epithelial and mesenchymal proteins from CPTAC. **a** t-SNE plots of tumor samples based on global proteome profile data from CPTAC. Each point represents a tumor-adjacent-normal pair. Each point is coloured by the tissue of origin (1), expression of epithelial proteins (2), or expression of mesenchymal proteins (3). **b** Average expression of epithelial proteins in tumor-adjacent-normal pair across cancer types from CPTAC data. **c** Average expression of mesenchymal proteins in tumor-adjacent-normal pair across cancer types from CPTAC data. **d** Average expression of epithelial genes in cell lines across cancer types from CCLE transcriptomics data. **e** Average expression of mesenchymal genes in cell lines across cancer types from CCLE transcriptomics data

To further assess the EMT-related protein expression in clinical samples and understand how these proteins are associated with oncogenic transformation across different tumor types, we analyzed quantitative proteomics data from the CPTAC repository [41]. Quantitative proteomics data for 539 cases with tumor-normal paired samples across breast and colorectal carcinoma, ovarian cancer, clear cell renal cell carcinoma (ccRCC), lung adenocarcinoma and, uterine corpus endometrial carcinoma (UCEC) were analyzed by the t-SNE method. We observed that irrespective of their oncogenic transformation, tumor samples were clustered based on their tissue of origin and retained their cellular and molecular identity (Fig. 5a, Left panel). These results are in concordance with transcriptome-based data from the CCLE and proteome based data by Koplev et al. on cell lines which further reinforces the recapitulation of tumor characteristics by cell line models [42]. While ccRCC tumors showed a distinct lower expression of epithelial proteins (Fig. 5a-middle panel) and overexpression of mesenchymal proteins (Fig. 5a-right panel), no such specific pattern was observed for other cancer types, although we also observed a sub-cluster of colorectal tumors with high epithelial and low mesenchymal protein expression pattern. This is further evident from the scatter plot showing the average expression values of epithelial or mesenchymal proteins across tumor samples for different cancer types (Fig. 5b, c). Similar results were also observed for renal cancer cell lines based on average expression values of epithelial or mesenchymal genes (Fig. 5d, e). These results indicate that EMT governs the cellular and molecular states of tumors across cancer types, and that ccRCC may be exceptionally prone to EMT.

## Discussion

EMT is a dynamic change in cellular architecture that leads to changes in cell migration and invasion. Its role has been well documented in developmental process and closely associated with tumor dissemination and metastasis [43, 44]. Several genetic, epigenetic, and proteomic regulators are known to coordinate this highly complex process. Various studies have reported the gain and loss of cellular protein components related with EMT. For example loss of expression of epithelial marker E-cadherin is regulated by differential expression of transcriptional repressors such as SNAI1/2, ZEB1/2,TWIST1/2 etc. [2]. A comprehensive study using the transcriptomics data by Tan et al. showed the interplay between EMT across cancer types [25]. They established a method to compute EMT score using published EMT signatures. Similar effort to define and predict EMT phenotype based on scoring matrices using transcriptomics data was published by Guo et al*.* [45]*,* and George et al*.* [46]. Another study by Mak et al. derived pan-cancer EMT gene signature that encompasses core EMT markers functioning across different tumors and calculated EMT score for 11 available distinct tumor types datasets [47]. However, these approaches lack the assessment of EMT at the protein level. In the present study, we aim to develop a method based on a targeted proteomics approach to assess the expression of a panel of EMT-related markers across different cancer types. We employed parallel reaction monitoring (PRM) based targeted proteomics strategy to quantify EMT markers. The established proteome panel and the targeted method in our study will help to monitor changes in EMT expression profile and characterization of tumor phenotype. PRM allows selective targeting of predefined precursor ions for fragmentation. Signal abundance of fragment ions indicates abundance of corresponding peptides in each sample. Proteotypic peptides from EMT markers were selectively targeted and monitored across samples. This strategy allowed quantification of EMT markers with high accuracy. To this effect, we curated a panel of 37 proteins belonging to molecular classes such as transcription factors, cytoskeletal proteins, and cell adhesion molecules. Gene ontology-based classification of biological processes associated with these proteins demonstrated that they are associated with EMT-related processes such as escape from programmed cell death, epithelial cell differentiation and cell migration etc. To the best of our knowledge, this is the first effort to define absolute quantification of the proteins involved in EMT event.

We have also analyzed pan-cancer transcriptomics data from 1037 cell lines in the CCLE database [30]. Organization of cells based on their transcriptome profile on t-SNE maps showed that the cell lines clustered largely according to their tissue of origin irrespective of the oncogenic transformation. Similar results were reported by Koplev et al*. *at both transcript and proteome levels [42]. A false coloured t-SNE map of cell lines based on epithelial or mesenchymal gene expression demonstrated that cell lines showing high expression of epithelial genes show a low expression of mesenchymal genes and vice versa. Besides, these cell lines are also organized in two distinct clusters based on the expression of either epithelial or mesenchymal genes alone. Koplev et al. have also demonstrated similar bimodal segregation of cell lines based on the expression of E-cadherin at both the protein and transcript level [42]. These results indicate that the expression of epithelial and mesenchymal genes play a deterministic role in defining cellular phenotype across cancer types, irrespective of the tissue of origin.

The advent of advanced high throughput proteomic techniques has made it possible to study cellular proteome in context to cellular plasticity. Since then it has been repeatedly noted that transcriptome and proteome abundances do not correlate adequately to be considered as proxies for each other [36–38]. The discordance of the data at transcriptome or at proteome levels could be because of the post-translational regulations of cellular proteins. However, large-scale proteomic data sets akin to the CCLE transcriptome data are not available for the expression of EMT-related proteins, to enable the study of their association with cellular phenotype and corresponding changes under different cellular contexts. Thus, effective methods to monitor changes in proteins related to EMT are needed to elucidate these cellular processes.

To this end, we have developed a PRM-based targeted proteomics method for the quantitative evaluation of several proteins related to EMT. We observed a higher abundance of epithelial phenotype-related proteins in known epithelial cell lines such as MCF7, Cal27 and FaDu along with a lower abundance of mesenchymal related proteins. Similarly, we observe a lower abundance of epithelial phenotype proteins in mesenchymal cell lines such as MDAMB231, J82 and UMUC3. These observations confirm that these cell lines generally exhibit a differentiating pattern of expression of EMT related proteins based on their cellular phenotype. Further, we observe a similar PCA-based clustering of both epithelial and mesenchymal cell lines into 2 distinct groups related to their phenotype and EMT scores with either transcriptome or PRM-based targeted proteomics data. This indicates that the PRM-based targeted proteomics data is largely concordant with the EMT scoring matrices that are based on transcriptomics.

Further, we observed clustering of gall bladder cancer cell lines (G415, NoZ, and OCUG1), which are not represented in the CCLE transcriptome database, with other mesenchymal cell lines. OCUG-1 and NOZ have been characterized as moderately invasive cell lines while G-415 has been characterized as highly invasive [48, 49]. In contrast, we observed clustering of the A549 lung adenocarcinoma cell line with epithelial cell lines in proteomics data and mesenchymal cell lines in transcriptomics data. Tan et al. has also assigned this cell line a score of 0.37 using their EMT scoring matrix indicating a mesenchymal phenotype. However, this cell line is known to be an epithelial cell line based on multiple reports of its non-invasive characteristics, along with the expression of epithelial markers such as E-cadherin [50, 51]. Our findings may underpin the propensity of these cells for EMT induction heterogeneity and plasticity associated with therapy resistance [52]. Further, we also observed clustering of SW780 and VMCUB1 cells, which had EMT scores of − 0.6 and − 0.23, respectively, with mesenchymal cell lines in the proteomics data. However, these cell lines clustered along with other epithelial cell lines based on transcriptomics data. Interestingly, SW780 and VMCUB1 have shown higher migration capability and a moderately invasive nature compared to RT112; an epithelial cell line [53]. Further, only VMCUB1 cell line has been reported to undergo EMT upon lentiviral transduction of HDAC5 or overexpression of lncRNA HOTAIR compared to other epithelial urinary bladder cancer cell lines such as RT112 and 5637 [54, 55]. Indicators of EMT are also observed in certain bladder cancers in vivo*,* including cancers progressing from basal-squamous molecular subtype exemplified by cell lines such as VMCUB1 [56]. Further, we observed a low correlation score between mRNA and protein expression in A549, VMCUB1 and SW780 cell lines compared to both epithelial (RT112 and MCF7) and mesenchymal (UMUC3 and MDAMB231) cell lines. Our observation thus reflects that certain subtle changes related to EMT might be more visible at the protein level and may be useful in complementing the insights available from other omics data.

Cytokeratins are structural proteins that enable cellular integrity. Downregulation of the KRT8/KRT18 keratin pair is known to induce an increase in cell motility and invasion [57]. We observed higher protein abundance of keratins 8 and 18 in epithelial cell lines RT112 and MCF7 compared to mesenchymal cell lines such as UMUC3 and MDAMB231. Further, we observed separate clustering of mesenchymal and epithelial cell lines for these keratins at the protein level but not at the mRNA level. We also observed discordance between proteins and mRNA abundance for SW780 and VMCUB1 cell lines relative to other epithelial cell lines such as RT112 and MCF7, where the protein abundance of these epithelial markers was more in line with the cellular phenotype of moderate invasiveness and higher migration capabilities. Similarly, we observed SW780 and VMCUB1 expressing higher abundance of the mesenchymal protein vimentin closer to the range shown by mesenchymal cell lines UMUC3 and J82. Interestingly, the mRNA abundance of vimentin in these cell lines is higher than other epithelial cell lines but lower than the mesenchymal cell lines. Our observation suggests the significance of quantitating protein abundances to predict the cellular plasticity with respect to the epithelial/mesenchymal/hybrid states.

To further explore how EMT-related proteins are expressed in clinical samples across multiple cancer types we analyzed quantitative proteomics data from the CPTAC database [41]. Based on the proteome profile, tumor samples from different cancers organized into tight clusters according to their tissue of origin, indicating that akin to cell line samples, tumor samples also retain their molecular and cellular identity irrespective of oncogenic transformation. Further, we observed that tumor samples were organized in distinct clusters based on the expression of epithelial and mesenchymal proteins. With respect to tissue of origin we observed that clear cell renal cell carcinoma (ccRCC) samples clustered in region with low expression of epithelial proteins and high expression of mesenchymal proteins based on the expression of epithelial and mesenchymal proteins respectively. Transcriptomics data from CCLE also showed that renal cancer cells show high mesenchymal gene expression and low epithelial gene expression. These results indicated a mesenchymal phenotype for renal carcinoma samples. Similarly, we observed that colorectal cancer tumor samples primarily clustered in the regions of high epithelial protein expression and low mesenchymal protein expression indicating an epithelial feature of these samples. Tan et al*.* has also reported a similar finding for colorectal cancer in terms of both tumor tissue and cell line samples, based on transcriptomics data. They have further hypothesized that these features of certain cancer types exhibiting epithelial or mesenchymal characteristics may be associated with embryonic ectodermal or mesodermal origins of these organs [25]. Thus, our analysis emphasizes the importance of proteomic analysis compared to the transcriptome-only approaches. Overall, we demonstrated that the expression of EMT-related genes is associated with the oncogenic transformation of cancer cells in both cell line models as well as tumor samples. We further showed that protein abundance data can be leveraged in addition to gene expression data to elucidate complex phenomena underlying EMT as well as its correlation with cancer progression and chemotherapeutic resistance. We believe the targeted proteomics strategy employed in our study can be used as a general-purpose tool for accurate estimation of EMT, and could be used to more accurately determine the impact of EMT effectors or drugs and assess changes in cellular phenotype.

## Materials and methods

### Bioinformatics and statistical analysis

The CCLE mRNA data and cell line annotations of 1037 cancer cell lines were retrieved from the CCLE portal at: https://portals.broadinstitute.org/ccle. To visualize the high-dimensional transcriptomics data we used the t-distributed stochastic neighbor embedding (t-SNE) algorithm implemented in the Rtsne package in R v.4.0.1 (http://www.R-project.org/) with perplexity value of 30 and at 1000 iterations, and all other arguments at their default values. Out of 18 cell lines that are used in the current study mRNA data for OCUG1, NoZ, G415 were not available in CCLE. For further analysis mRNA expression values of 20 selected proteins for 15 cell lines were used. Principal Component Analysis (PCA) was performed using the R-based “prcomp” function.

CCLE has profiled Reverse Phase Protein Array (RPPA) data for 159 proteins in 889 cell lines. Out of 18 cancer cell lines that are included in our study, 7 cell lines have both available RPPA and transcriptomic data. 59 proteins were profiled in common between CCLE RPPA data and CCLE transcriptomic data. Pearson's correlation coefficient (r) was used to evaluate the relationship between mRNA expression and protein expression in cancer cell lines.

To analyze the expression of EMT related protein in cancer tissue samples we used publicly available quantitative proteomics data from Clinical Proteomic Tumor Analysis Consortium (CPTAC) repository (https://cptac-data-portal.georgetown.edu/cptacPublic/) [40]. Proteomics data of six cancer types including breast cancer, clear cell renal cell carcinoma (CCRCC), colon cancer, lung adenocarcinoma (LUAD), ovarian cancer, and uterine corpus endometrial carcinoma (UCEC) were downloaded from the CPTAC data portal. Samples with matched tumor and normal data were taken for further analysis whereas the unmatched samples were filtered out. Within each proteomics dataset we applied z-score normalization of logged expression values to all the samples. To visualize the proteomics data we again used the t-SNE algorithm with the same parameters as mentioned above. Out of 20 selected proteins, 15 proteins were quantified in all six CPTAC proteomics data. For plotting scatter plot, we averaged the expression of selected epithelial and mesenchymal related proteins separately for individual samples in each cancer type.

### Cell culture conditions

Cell lines used in the current study were grown as per vendor recommendations. Briefly the cells were cultured in their respective media as detailed in Additional file 1: Method S1 and 1% penicillin/streptomycin mixture at 37 °C in a humidified 5% CO_2_ atmosphere. Cells were harvested at 70% confluency. Gall bladder cancer cell lines G-415 was sourced from RIKEN Bio Resource Center, Ibaraki, Japan and OCUG-1 and NOZ from Health Science Research Resources Bank, Osaka, Japan. Bladder cancer cell lines were received from Prof. Jean Paul Thiery (Department of Biochemistry, National University of Singapore, Singapore).

### Trypsin digestion and Sep-Pak C_18_ column-based cleanup

All the cell lines were grown in recommended media, and prior to harvesting washed with ice-cold phosphate-buffered saline thrice to remove media residuals. Cells were then harvested and lysed in urea lysis buffer (20 mM HEPES pH 8.0, 9 M urea, 1 mM sodium orthovanadate, 2.5 mM sodium pyrophosphate, 1 mM phosphoglycerophosphate). Protein concentration was measured using the bicinchoninic acid assay method as per the manufacturer’s protocol (Thermo Scientific, Bremen, Germany). 500 µg equivalent of protein from each sample were reduced using dithiothreitol (DTT, 5 mM) at 60 °C for 20 min and alkylated with iodoacetamide (IAA, 20 mM) for 10 min at room temperature. Protein was precipitated overnight at − 80 °C using ice-cold acetone. The samples were centrifuged at 12,000 rpm for 15 min; the acetone was removed and the pellet air-dried and then dissolved in 4 M urea. Proteins were then digested using lysyl endopeptidase, Mass Spectrometry Grade (catalog no. 125–05061; Wako, Richmond, VA) at 1:100 enzyme to protein ratio for 4 h at 37 °C. After 4 h, the urea concentration was reduced from 4 to 2 M using 50 mM Triethylammonium bicarbonate (TEABC). The samples were then digested using tosyl phenylalanyl chloromethyl ketone (TPCK)-treated trypsin (Worthington, NJ) at a 1:20 enzyme to protein ratio for 16 h at 37 °C. The samples were cleaned using Sep-Pak Classic C18 columns (catalog no. WAT051910; Waters, Milford, MA) and then completely dried before LC–MS/MS analysis.

### LC–MS/MS method

The peptides were analyzed on a QExactive plus mass spectrometer interfaced with RS-nanoLC 3000 nanoflow liquid chromatography system (Thermo Scientific, Bremen, Germany). 5 µg equivalent peptide digests were reconstituted in 0.1% formic acid and loaded onto a trap column (Thermo Scientific™ Acclaim™ PepMap™ 100 C18, 75 µm × 2 cm, 3 µm particle size, 100 Å pore size) at a flow rate of 5 µl/min and resolved on analytical column (Thermo Scientific™ EASY-Spray™ C18 2 µm particle size, 100 Å pore size, 75µmx50cm) at a flow rate of 300 nl/min. The peptides were resolved using a step gradient of 5–25% solvent B (0.1% formic acid in 85% acetonitrile) from 8 to 60 min and 25–40% solvent B for 60–85 min. The mass spectrometer was operated in data-independent acquisition PRM mode. A survey full scan MS (from m/z 350–1700) was acquired in the Orbitrap at a resolution of 70,000 at 200 m/z. A targeted list of precursor ions with charge state ≥ 2 was isolated and fragmented using HCD fragmentation with 32% normalized collision energy and detected at a mass resolution of 30,000 at 200 m/z. The data were subsequently analyzed using Skyline [58].

### Optimization of PRM assay

The PRM assay for a selected panel of 37 epithelial and mesenchymal proteins as represented in Additional file 1: Fig. S1 was developed by selecting proteotypic peptides based on standard criteria for targeted proteomics [29, 33–35]. A list of 116 proteotypic peptides selected for analysis is represented in Additional file 2: Table S1. For the development of assay, 100 femtomol of each stable isotope-labelled peptide and its synthetic light version was subjected to data-dependent MS/MS analysis. From this analysis consistently detected peptides were considered as detectable targets. A time scheduled PRM method for 31 target proteins with 96 peptides was developed. The lower limit of detection, the lower limit of quantitation, and the linear range were assessed using a reverse calibration curve strategy. We spiked 500 femtomol of synthetic light version of the selected target peptides (for normalization) and varied the amount of heavy isotope-labelled peptide in 1 µg of pooled cell lysate (100 attomol to 1 picomol). A concentration curve was generated by taking the ratio of SIL/light peptides and the lower limit of detection (LOD) and quantitation (LOQ) was estimated using the Skyline software. Linear regression analysis in log10 space was performed with a maximum LOQ bias of 10% and LOQ CV of 20%. As lower limit of quantitation for few peptides was 20 femtomol, for PRM analysis 25 femtomol equivalent of heavy peptide mix was spiked per 5 µg of cell line protein digest.

### Data processing

All PRM-MS raw files were processed in Skyline to generate XIC and perform peak integration [58]. We assessed the PRM data for (a) peak symmetry (b) endogenous peptide and SIL peptide retention time alignment (c) retention time alignment across transitions for peptides [59]. Although we detected multiple peptides for each protein, based on quality control criteria and consistent detection across cell lines, 31 best peptides corresponding to 31 proteins (Additional file 1: Fig. S2 and 3, Additional file 3: Table S2) were selected for quantitation. The summed peak area of at least 3 most intense fragment ions was used to quantify the endogenous and heavy peptides, respectively. To determine the relative abundance of the target peptides, the summed peak area of endogenous peptides was first normalized to their corresponding heavy standards. Thus, the relative expression level of each peptide in the sample was calculated as the ratio of the signal intensities between the light peptide (endogenous) and heavy peptide (L/H ratio). A set of 20 proteins with at least 100 attomole protein concentration were further filtered.

## Supplementary Information

**Additional file 1:** **Figure S1.** Gene ontology-based annotation of proteins used for PRM-based proteomics analysis curated from literature. **Figure S2.** PRM transitions for 31 peptides precursors selected for analysis of 31 proteins mentioned in the figures across the cell lines. **Figure S3.** Calibration curve for 31 peptides precursors selected for monitoring protein expression across cell lines. **Figure S4.** Co-relation plots for mRNA-based gene expression and protein expression for values for depicted cell lines based on transcriptomics and reversed phase protein array (RPPA) proteomics data from CCLE database. **Method S1.** The overview of all the cell lines used in the study along with the tissue of origin and culture method.

**Additional file 2: Table S1.** List of EMT-related proteins and peptides selected for PRM assays development.

**Additional file 3: Table S2.** List of EMT-related proteins and limit of detection and limit of quantitation values.

**Additional file 4: Table S3.** Relative expression of Epithelial and mesenchymal marker proteins across cell lines.

## Data Availability

Not applicable.

## Abbreviations

EMT: Epithelial-mesenchymal transition
PRM: Parallel reaction monitoring
tSNE: t-Distributed Stochastic Neighbor Embedding
CCLE: Cancer Cell Line Encyclopedia
TEABC: Triethylammonium bicarbonate
ACN: Acetonitrile
BCA: Bicinchoninic acid
AGC: Automatic gain control
HCD: Higher energy collisional dissociation
RIPA: Radioimmunoprecipitation assay buffer

## References

[1] Skovierova H, Okajcekova T, Strnadel J, Vidomanova E, Halasova E (2018). Molecular regulation of epithelial-to-mesenchymal transition in tumorigenesis (review). Int J Mol Med.

[2] Serrano-Gomez SJ, Maziveyi M, Alahari SK (2016). Regulation of epithelial–mesenchymal transition through epigenetic and post-translational modifications. Mol Cancer.

[3] Raja R, Pandey A, Kumar P (2020). Epithelial to mesenchymal plasticity: role in cancer progression. Front Biosci (Landmark Ed).

[4] Saitoh M (2018). Involvement of partial EMT in cancer progression. J Biochem.

[5] Sha Y, Haensel D, Gutierrez G, Du H, Dai X, Nie Q (2019). Intermediate cell states in epithelial-to-mesenchymal transition. Phys Biol.

[6] Pastushenko I, Brisebarre A, Sifrim A (2018). Identification of the tumour transition states occurring during EMT. Nature.

[7] Yadavalli S, Jayaram S, Manda SS (2017). Data-driven discovery of extravasation pathway in circulating tumor cells. Sci Rep.

[8] De T, Goyal S, Balachander G, Chatterjee K, Kumar P, Babu KG, Rangarajan A (2019). A novel ex vivo system using 3D polymer scaffold to culture circulating tumor cells from breast cancer patients exhibits dynamic E-M phenotypes. J Clin Med.

[9] Williams ED, Gao D, Redfern A, Thompson EW (2019). Controversies around epithelial–mesenchymal plasticity in cancer metastasis. Nat Rev Cancer.

[10] Stemmler MP, Eccles RL, Brabletz S, Brabletz T (2019). Non-redundant functions of EMT transcription factors. Nat Cell Biol.

[11] Nieto MA (2002). The snail superfamily of zinc-finger transcription factors. Nat Rev Mol Cell Biol.

[12] Yang J, Antin P, Berx G (2020). Guidelines and definitions for research on epithelial–mesenchymal transition. Nat Rev Mol Cell Biol.

[13] Lourenco AR, Ban Y, Crowley MJ (2020). Differential contributions of pre- and post-EMT tumor cells in breast cancer metastasis. Cancer Res.

[14] Song J, Wang W, Wang Y, Qin Y, Wang Y, Zhou J, Wang X, Zhang Y, Wang Q (2019). Epithelial–mesenchymal transition markers screened in a cell-based model and validated in lung adenocarcinoma. BMC Cancer.

[15] Puram SV, Tirosh I, Parikh AS (2017). Single-cell transcriptomic analysis of primary and metastatic tumor ecosystems in head and neck cancer. Cell.

[16] Anastassiou D, Rumjantseva V, Cheng W, Huang J, Canoll PD, Yamashiro DJ, Kandel JJ (2011). Human cancer cells express Slug-based epithelial–mesenchymal transition gene expression signature obtained in vivo. BMC Cancer.

[17] Edfors F, Danielsson F, Hallstrom BM, Kall L, Lundberg E, Ponten F, Forsstrom B, Uhlen M (2016). Gene-specific correlation of RNA and protein levels in human cells and tissues. Mol Syst Biol.

[18] Liu Y, Beyer A, Aebersold R (2016). On the dependency of cellular protein levels on mRNA abundance. Cell.

[19] de Sousa AR, Penalva LO, Marcotte EM, Vogel C (2009). Global signatures of protein and mRNA expression levels. Mol Biosyst.

[20] Silvestrini VC, Lanfredi GP, Masson AP, Poersch A, Ferreira GA, Thome CH, Faca VM (2019). A proteomics outlook towards the elucidation of epithelial–mesenchymal transition molecular events. Mol Omics.

[21] Vergara D, Simeone P, Franck J (2016). Translating epithelial mesenchymal transition markers into the clinic: novel insights from proteomics. EuPA Open Proteom.

[22] Smolders K, Lombaert N, Valkenborg D, Baggerman G, Arckens L (2015). An effective plasma membrane proteomics approach for small tissue samples. Sci Rep.

[23] Jiang D, Jarrett HW, Haskins WE (2009). Methods for proteomic analysis of transcription factors. J Chromatogr A.

[24] Simicevic J, Deplancke B (2017). Transcription factor proteomics—tools, applications, and challenges. Proteomics.

[25] Tan TZ, Miow QH, Miki Y, Noda T, Mori S, Huang RY, Thiery JP (2014). Epithelial-mesenchymal transition spectrum quantification and its efficacy in deciphering survival and drug responses of cancer patients. EMBO Mol Med.

[26] Priyanka Chakraborty JTG, Tripathi S, Levine H, Jolly MK (2020). Comparative study of transcriptomics-based scoring metrics for the epithelial-hybrid-mesenchymal spectrum. Front Bioeng Biotechnol.

[27] Kim YJ, Gallien S, van Oostrum J, Domon B (2013). Targeted proteomics strategy applied to biomarker evaluation. Proteomics Clin Appl.

[28] Peterson AC, Russell JD, Bailey DJ, Westphall MS, Coon JJ (2012). Parallel reaction monitoring for high resolution and high mass accuracy quantitative, targeted proteomics. Mol Cell Proteomics.

[29] Lange V, Picotti P, Domon B, Aebersold R (2008). Selected reaction monitoring for quantitative proteomics: a tutorial. Mol Syst Biol.

[30] Ghandi M, Huang FW, Jane-Valbuena J (2019). Next-generation characterization of the cancer cell line encyclopedia. Nature.

[31] Van der Maaten L, Hinton G (2008). Visualizing data using t-SNE. J Mach Learn Res.

[32] Sathe G, Mangalaparthi KK, Jain A, Darrow J, Troncoso J, Albert M, Moghekar A, Pandey A (2020). Multiplexed phosphoproteomic study of brain in patients with Alzheimer's disease and age-matched cognitively healthy controls. OMICS.

[33] Gallien S, Bourmaud A, Kim SY, Domon B (2014). Technical considerations for large-scale parallel reaction monitoring analysis. J Proteomics.

[34] Liebler DC, Zimmerman LJ (2013). Targeted quantitation of proteins by mass spectrometry. Biochemistry.

[35] Picotti P, Rinner O, Stallmach R, Dautel F, Farrah T, Domon B, Wenschuh H, Aebersold R (2010). High-throughput generation of selected reaction-monitoring assays for proteins and proteomes. Nat Methods.

[36] Vogel C, Marcotte EM (2012). Insights into the regulation of protein abundance from proteomic and transcriptomic analyses. Nat Rev Genet.

[37] Nagaraj N, Wisniewski JR, Geiger T, Cox J, Kircher M, Kelso J, Paabo S, Mann M (2011). Deep proteome and transcriptome mapping of a human cancer cell line. Mol Syst Biol.

[38] Le Roch KG, Johnson JR, Florens L (2004). Global analysis of transcript and protein levels across the *Plasmodium**falciparum* life cycle. Genome Res.

[39] McCorry AM, Loughrey MB, Longley DB, Lawler M, Dunne PD (2018). Epithelial-to-mesenchymal transition signature assessment in colorectal cancer quantifies tumour stromal content rather than true transition. J Pathol.

[40] Byers LA, Diao L, Wang J (2013). An epithelial-mesenchymal transition gene signature predicts resistance to EGFR and PI3K inhibitors and identifies Axl as a therapeutic target for overcoming EGFR inhibitor resistance. Clin Cancer Res.

[41] Edwards NJ, Oberti M, Thangudu RR, Cai S, McGarvey PB, Jacob S, Madhavan S, Ketchum KA (2015). The CPTAC data portal: a resource for cancer proteomics research. J Proteome Res.

[42] Koplev S, Lin K, Dohlman AB, Ma'ayan A (2018). Integration of pan-cancer transcriptomics with RPPA proteomics reveals mechanisms of epithelial–mesenchymal transition. PLoS Comput Biol.

[43] Jain AP, Patel K, Pinto S (2019). MAP2K1 is a potential therapeutic target in erlotinib resistant head and neck squamous cell carcinoma. Sci Rep.

[44] Dongre A, Weinberg RA (2019). New insights into the mechanisms of epithelial–mesenchymal transition and implications for cancer. Nat Rev Mol Cell Biol.

[45] Guo CC, Majewski T, Zhang L (2019). Dysregulation of EMT drives the progression to clinically aggressive sarcomatoid bladder cancer. Cell Rep.

[46] George JT, Jolly MK, Xu S, Somarelli JA, Levine H (2017). Survival outcomes in cancer patients predicted by a partial EMT gene expression scoring metric. Cancer Res.

[47] Mak PM, Tong P, Diao L (2016). A patient-derived, pan-cancer EMT signature identifies global molecular alterations and immune target enrichment following epithelial-to-mesenchymal transition. Clin Cancer Res.

[48] Gondkar K, Patel K, Patil Okaly GV, Nair B, Pandey A, Gowda H, Kumar P (2019). Dickkopf Homolog 3 (DKK3) acts as a potential tumor suppressor in gallbladder cancer. Front Oncol.

[49] Subbannayya T, Leal-Rojas P, Barbhuiya MA (2015). Macrophage migration inhibitory factor—a therapeutic target in gallbladder cancer. BMC Cancer.

[50] Lee HJ, Park MK, Lee EJ, Lee CH (2013). Resolvin D1 inhibits TGF-beta1-induced epithelial mesenchymal transition of A549 lung cancer cells via lipoxin A4 receptor/formyl peptide receptor 2 and GPR32. Int J Biochem Cell Biol.

[51] Tirino V, Camerlingo R, Bifulco K (2013). TGF-beta1 exposure induces epithelial to mesenchymal transition both in CSCs and non-CSCs of the A549 cell line, leading to an increase of migration ability in the CD133+ A549 cell fraction. Cell Death Dis.

[52] Tieche CC, Gao Y, Buhrer ED (2019). Tumor initiation capacity and therapy resistance are differential features of EMT-related subpopulations in the NSCLC cell line A549. Neoplasia.

[53] Deb B, Puttamallesh VN, Gondkar K, Thiery JP, Gowda H, Kumar P (2019). Phosphoproteomic profiling identifies aberrant activation of integrin signaling in aggressive non-type bladder carcinoma. J Clin Med.

[54] Jaguva Vasudevan AA, Hoffmann MJ, Beck MLC (2019). HDAC5 expression in urothelial carcinoma cell lines inhibits long-term proliferation but can promote epithelial-to-mesenchymal transition. Int J Mol Sci.

[55] Heubach J, Monsior J, Deenen R, Niegisch G, Szarvas T, Niedworok C, Schulz WA, Hoffmann MJ (2015). The long noncoding RNA HOTAIR has tissue and cell type-dependent effects on HOX gene expression and phenotype of urothelial cancer cells. Mol Cancer.

[56] Earl J, Rico D, Carrillo-de-Santa-Pau E (2015). The UBC-40 urothelial bladder cancer cell line index: a genomic resource for functional studies. BMC Genomics.

[57] Fortier AM, Asselin E, Cadrin M (2013). Keratin 8 and 18 loss in epithelial cancer cells increases collective cell migration and cisplatin sensitivity through claudin1 up-regulation. J Biol Chem.

[58] MacLean B, Tomazela DM, Shulman N (2010). Skyline: an open source document editor for creating and analyzing targeted proteomics experiments. Bioinformatics.

[59] Abbatiello S, Ackermann BL, Borchers C (2017). New guidelines for publication of manuscripts describing development and application of targeted mass spectrometry measurements of peptides and proteins. Mol Cell Proteomics.

