# Supplementary material for: Pan-cancer quantitation of epithelial-mesenchymal transition dynamics using parallel reaction monitoring-based targeted proteomics approach
Source: J Transl Med. 2022 Feb 11;20:84. doi: 10.1186/s12967-021-03227-0 (PMC8832824; doi:10.1186/s12967-021-03227-0)
Supplement: Supplementary file 1 — Additional file 1: Figure S1. Gene ontology-based annotation of proteins used for PRM-based proteomics analysis curated from literature. Figure S2. PRM transitions for 31 peptides precursors selected for analysis of 31 proteins mentioned in the figures across the cell lines. Figure S3. Calibration curve for 31 peptides precursors selected for monitoring protein expression across cell lines. Figure S4. Co-relation plots for mRNA-based gene expression and protein expression for values for depicted cell lines based on transcriptomics and reversed phase protein array (RPPA) proteomics data from CCLE database. Method S1. The overview of all the cell lines used in the study along with the tissue of origin and culture method. [file 12967_2021_3227_MOESM1_ESM.pdf]

Jain *et al.*, 2021. Pan-cancer quantitation of epithelial-mesenchymal transition dynamics using parallel reaction monitoring-based targeted proteomics approach

Supplementary Fig. 1 Gene ontology-based annotation of proteins used for PRM-based proteomics analysis curated from literature

Biological process	ANXA4	ANXA9	CDH1	DSG2	DSG3	DSP	EPCAM	FOXA1	FOXA2	ITGB4	ITGB6	KRT18	KRT8	MUC1	S100A14	SCNN1A	SDC1	XBP1	COL14A1	COL6A1	COL6A2	CDH2	EVI2A	FN1	ITGA5	LAMC1	LAMC2	SDC2	SNAI1	SNAI2	TWIST1	TWIST2	TUBA1A	VIM	WIPF1	ZEB1	ZEB2
Gene Association (Epithelial/ Mesenchymal)	E	E	E	E	E	E	E	E	E	E	E	E	E	E	E	E	E	E	M	M	M	M	M	M	M	M	M	M	M	M	M	M	M	M	M	M	M
Cell communication	■	■	■	■		■	■	■	■	■	■	■	■			■	■					■		■	■			■	■	■	■			■		■	■
Cell surface receptor signaling pathway	■							■		■	■	■	■			■	■					■	■	■	■			■	■	■	■			■		■	■
Positive regulation of cellular metabolic process			■				■	■	■													■		■	■				■	■	■			■		■	■
Cellular macromolecule metabolic process	■		■																			■						■	■	■	■	■		■		■	■
Cell development	■	■	■														■					■						■		■	■			■		■	■
Cell differentiation	■		■	■	■	■	■	■	■	■		■	■				■		■	■	■	■		■	■	■	■	■	■	■	■	■		■		■	■
Tissue development	■			■	■	■	■	■	■	■		■	■				■		■	■	■	■		■	■	■	■		■	■	■			■	■	■	■
Epithelium development	■			■	■	■	■	■				■	■				■					■			■				■	■	■			■		■	■
Mesenchymal cell differentiation								■	■															■					■	■	■						■
Extracellular matrix organization			■							■	■													■	■	■	■										
Cell migration			■							■	■				■		■					■		■	■	■	■	■	■	■	■	■					
Cell-cell adhesion		■	■					■	■	■	■	■		■								■		■	■	■	■			■							
Cell junction organization			■	■		■				■												■		■	■	■	■		■	■			■				
Regulation of cell motility			■				■								■			■						■	■		■		■	■	■	■					
Structural molecule activity						■						■							■	■	■			■		■	■						■	■			
Cellular component organization or biogenesis			■	■		■	■	■	■	■	■	■	■	■	■		■	■	■	■	■	■		■	■	■	■	■	■	■	■	■		■	■		■
Programmed cell death	■			■	■	■	■	■				■	■	■	■										■				■	■	■	■					
Regulation of apoptotic process	■						■	■				■		■				■							■				■	■	■	■					
Regulation of transcription by RNA polymerase II	■						■	■	■																				■	■	■	■				■	■
Positive regulation of gene expression			■				■	■	■															■					■		■			■		■	■

Supplementary Fig. 2 PRM transitions for 31 peptides precursors selected for analysis of 31 proteins mentioned in the figures across the cell lines

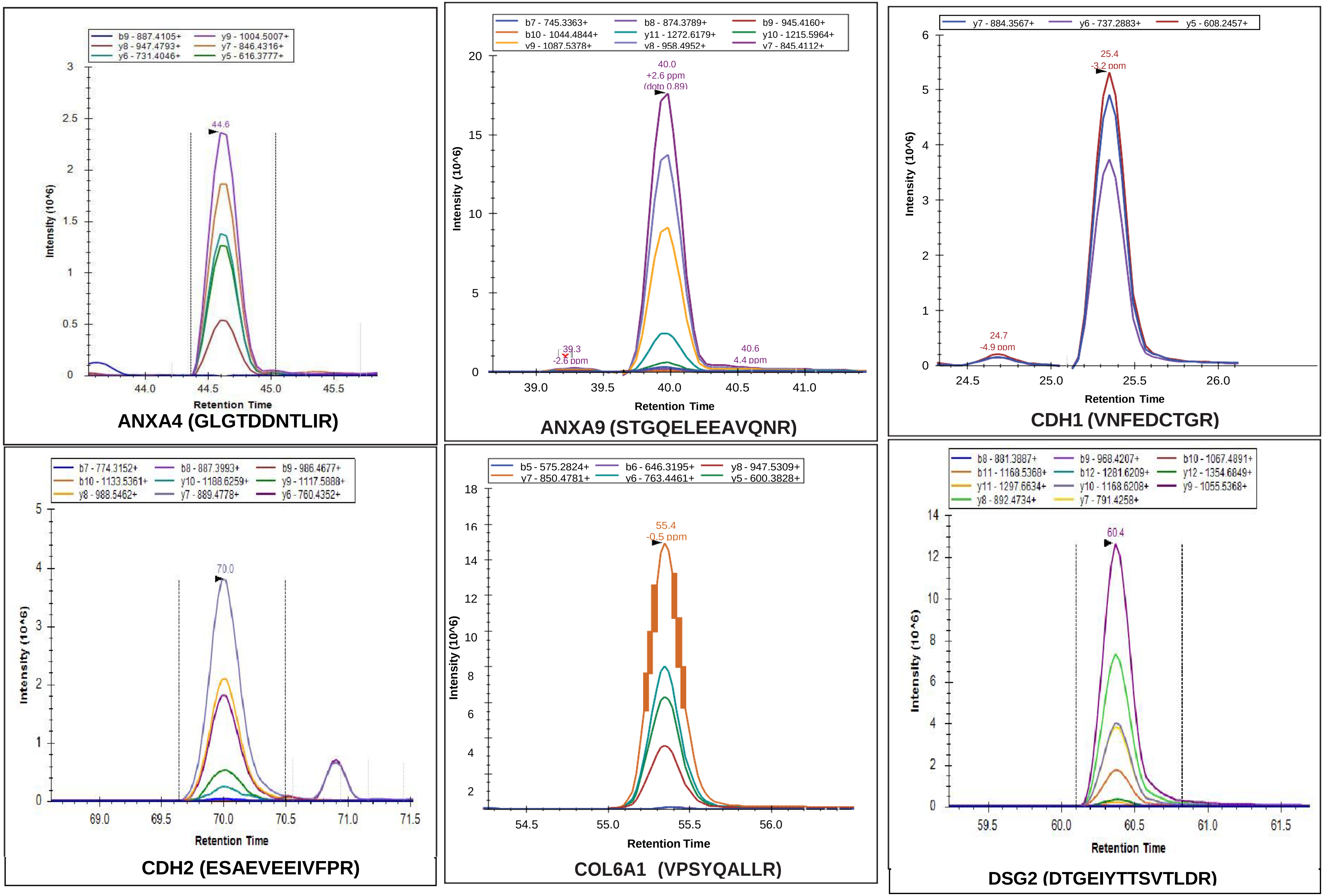

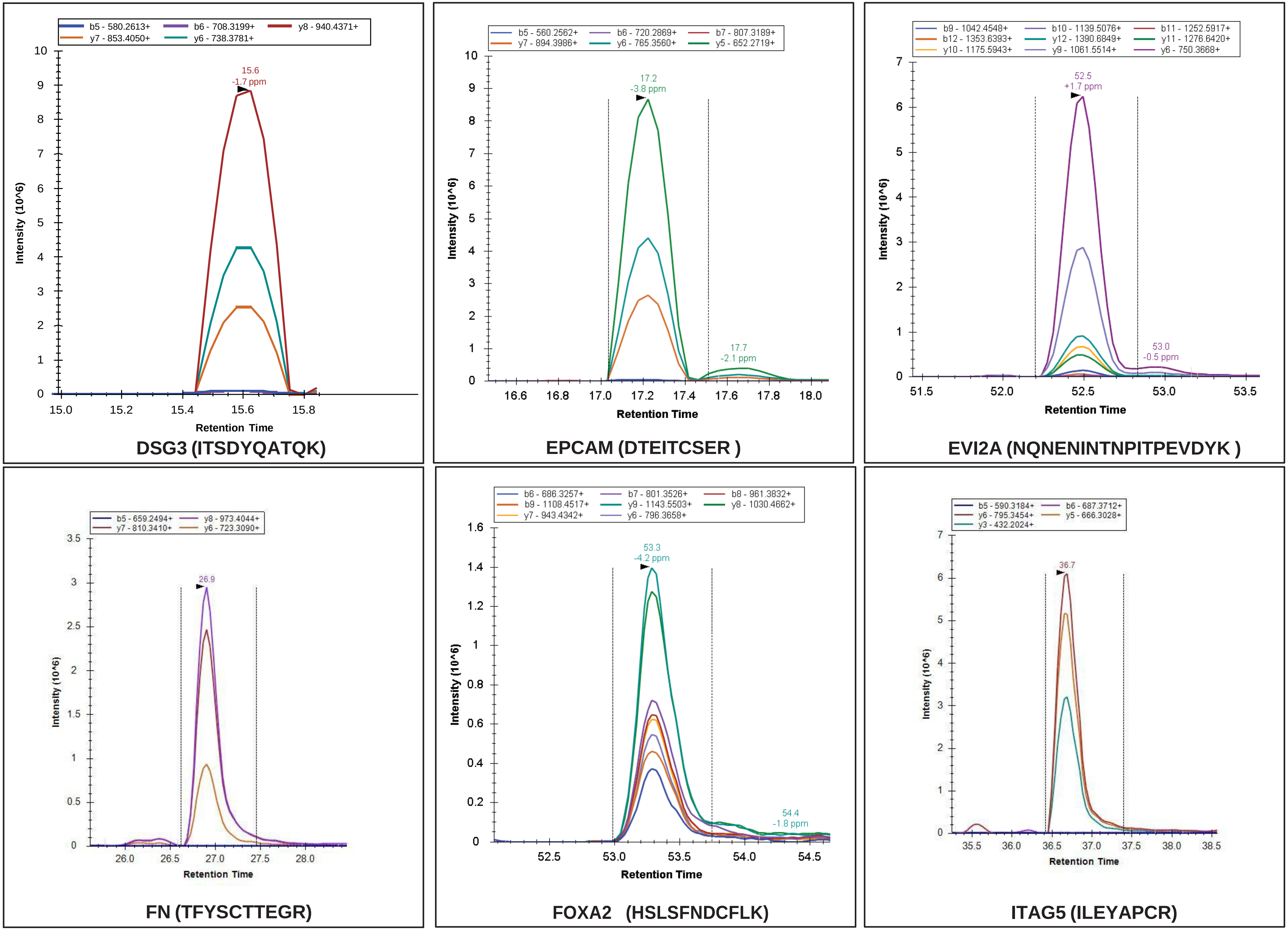

b5 - 580.2613+
b6 - 708.3199+
y8 - 940.4371+
y7 - 853.4050+
y6 - 738.3781+
15.6
-1.7 ppm
Intensity (10^6)
Retention Time
DSG3 (ITSDYQATQK)
b5 - 560.2562+
b6 - 720.2869+
b7 - 807.3189+
y7 - 894.3986+
y6 - 765.3560+
y5 - 652.2719+
17.2
-3.8 ppm
17.7
-2.1 ppm
Intensity (10^6)
Retention Time
EPCAM (DTEITCSER)
b9 - 1042.4548+
b10 - 1139.5076+
b11 - 1252.5917+
b12 - 1353.6393+
y12 - 1390.6849+
y11 - 1276.6420+
y10 - 1175.5943+
y9 - 1061.5514+
y6 - 750.3668+
52.5
+1.7 ppm
53.0
-0.5 ppm
Intensity (10^6)
Retention Time
EVI2A (NQNENINTNPITPEVDYK)
b5 - 659.2494+
y8 - 973.4044+
y7 - 810.3410+
y6 - 723.3090+
26.9
Intensity (10^6)
Retention Time
FN (TFYSCTTEGR)
b6 - 686.3257+
b7 - 801.3526+
b8 - 961.3832+
b9 - 1108.4517+
y9 - 1143.5503+
y8 - 1030.4662+
y7 - 943.4342+
y6 - 796.3658+
53.3
-4.2 ppm
54.4
-1.8 ppm
Intensity (10^6)
Retention Time
FOXA2 (HSLSFNDCFLK)
b5 - 590.3184+
b6 - 687.3712+
y6 - 795.3454+
y5 - 666.3028+
y3 - 432.2024+
36.7
Intensity (10^6)
Retention Time
ITAG5 (ILEYAPCR)

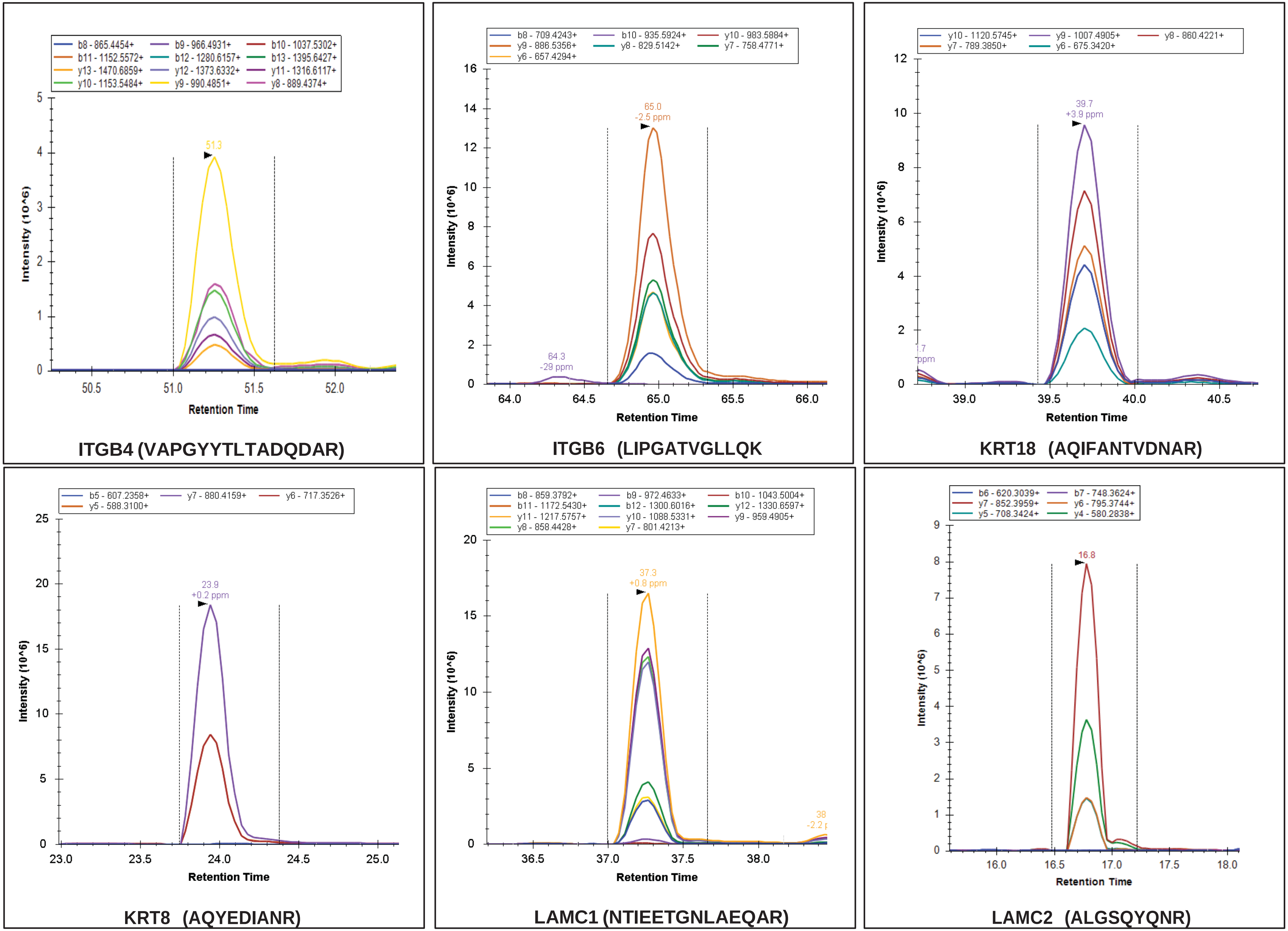

b8 - 865.4454+
b9 - 966.4931+
b10 - 1037.5302+
b11 - 1152.5572+
b12 - 1280.6157+
b13 - 1395.6427+
y13 - 1470.6859+
y12 - 1373.6332+
y11 - 1316.6117+
y10 - 1153.5484+
y9 - 990.4851+
y8 - 889.4374+
51.3
Intensity (10^6)
Retention Time
ITGB4 (VAPGYYTLTADQDAR)
b8 - 709.4243+
b10 - 935.5924+
y10 - 983.5884+
y9 - 886.5356+
y8 - 829.5142+
y7 - 758.4771+
y6 - 657.4294+
65.0
-2.5 ppm
64.3
-29 ppm
Intensity (10^6)
Retention Time
ITGB6 (LIPGATVGLLQK)
y10 - 1120.5745+
y9 - 1007.4905+
y8 - 860.4221+
y7 - 789.3850+
y6 - 675.3420+
39.7
+3.9 ppm
Intensity (10^6)
Retention Time
KRT18 (AQIFANTVDNAR)
b5 - 607.2358+
y7 - 880.4159+
y6 - 717.3526+
y5 - 588.3100+
23.9
+0.2 ppm
Intensity (10^6)
Retention Time
KRT8 (AQYEDIANR)
b8 - 859.3792+
b9 - 972.4633+
b10 - 1043.5004+
b11 - 1172.5430+
b12 - 1300.6016+
y12 - 1330.6597+
y11 - 1217.5757+
y10 - 1088.5331+
y9 - 959.4905+
y8 - 858.4428+
y7 - 801.4213+
37.3
+0.8 ppm
Intensity (10^6)
Retention Time
LAMC1 (NTIEETGNLAEQAR)
b6 - 620.3039+
b7 - 748.3624+
y7 - 852.3959+
y6 - 795.3744+
y5 - 708.3424+
y4 - 580.2838+
16.8
Intensity (10^6)
Retention Time
LAMC2 (ALGSQYQNR)

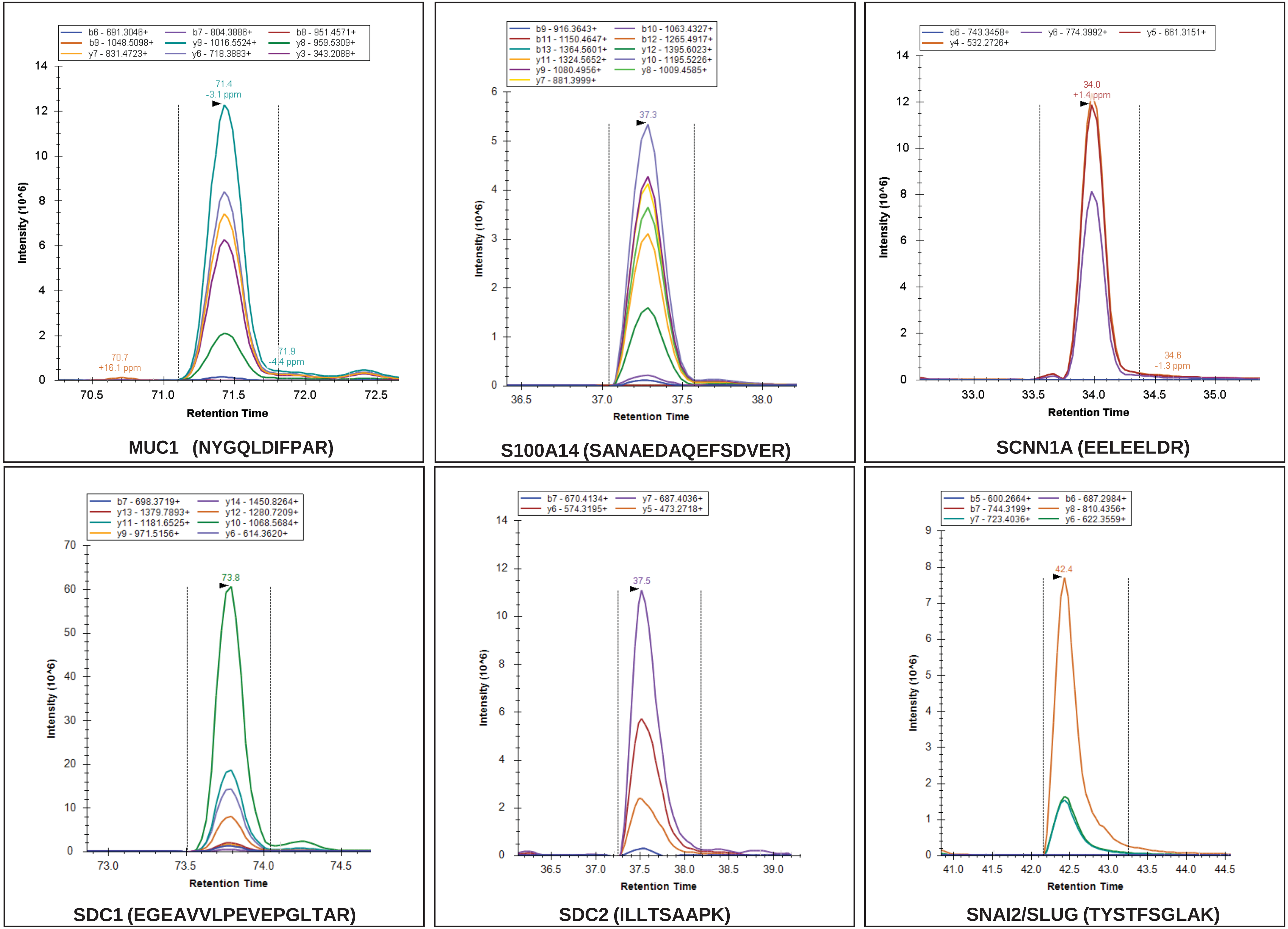
b6 - 691.3046+
b7 - 804.3886+
b8 - 951.4571+
b9 - 1048.5098+
y9 - 1016.5524+
y8 - 959.5309+
y7 - 831.4723+
y6 - 718.3883+
y3 - 343.2088+
71.4
-3.1 ppm
70.7
+16.1 ppm
71.9
-4.4 ppm
Intensity (10^6)
Retention Time
MUC1 (NYGQLDIFPAR)
b9 - 916.3643+
b10 - 1063.4327+
b11 - 1150.4647+
b12 - 1265.4917+
b13 - 1364.5601+
y12 - 1395.6023+
y11 - 1324.5652+
y10 - 1195.5226+
y9 - 1080.4956+
y8 - 1009.4585+
y7 - 881.3999+
37.3
Intensity (10^6)
Retention Time
S100A14 (SANAEDAQEFSDVER)
b6 - 743.3458+
y6 - 774.3992+
y5 - 661.3151+
y4 - 532.2726+
34.0
+1.4 ppm
34.6
-1.3 ppm
Intensity (10^6)
Retention Time
SCNN1A (EELEELDR)
b7 - 698.3719+
y14 - 1450.8264+
y13 - 1379.7893+
y12 - 1280.7209+
y11 - 1181.6525+
y10 - 1068.5684+
y9 - 971.5156+
y6 - 614.3620+
73.8
Intensity (10^6)
Retention Time
SDC1 (EGEAVVLPEVEPGLTAR)
b7 - 670.4134+
y7 - 687.4036+
y6 - 574.3195+
y5 - 473.2718+
37.5
Intensity (10^6)
Retention Time
SDC2 (ILLTSAAPK)
b5 - 600.2664+
b6 - 687.2984+
b7 - 744.3199+
y8 - 810.4356+
y7 - 723.4036+
y6 - 622.3559+
42.4
Intensity (10^6)
Retention Time
SNAI2/SLUG (TYSTFSGLAK)

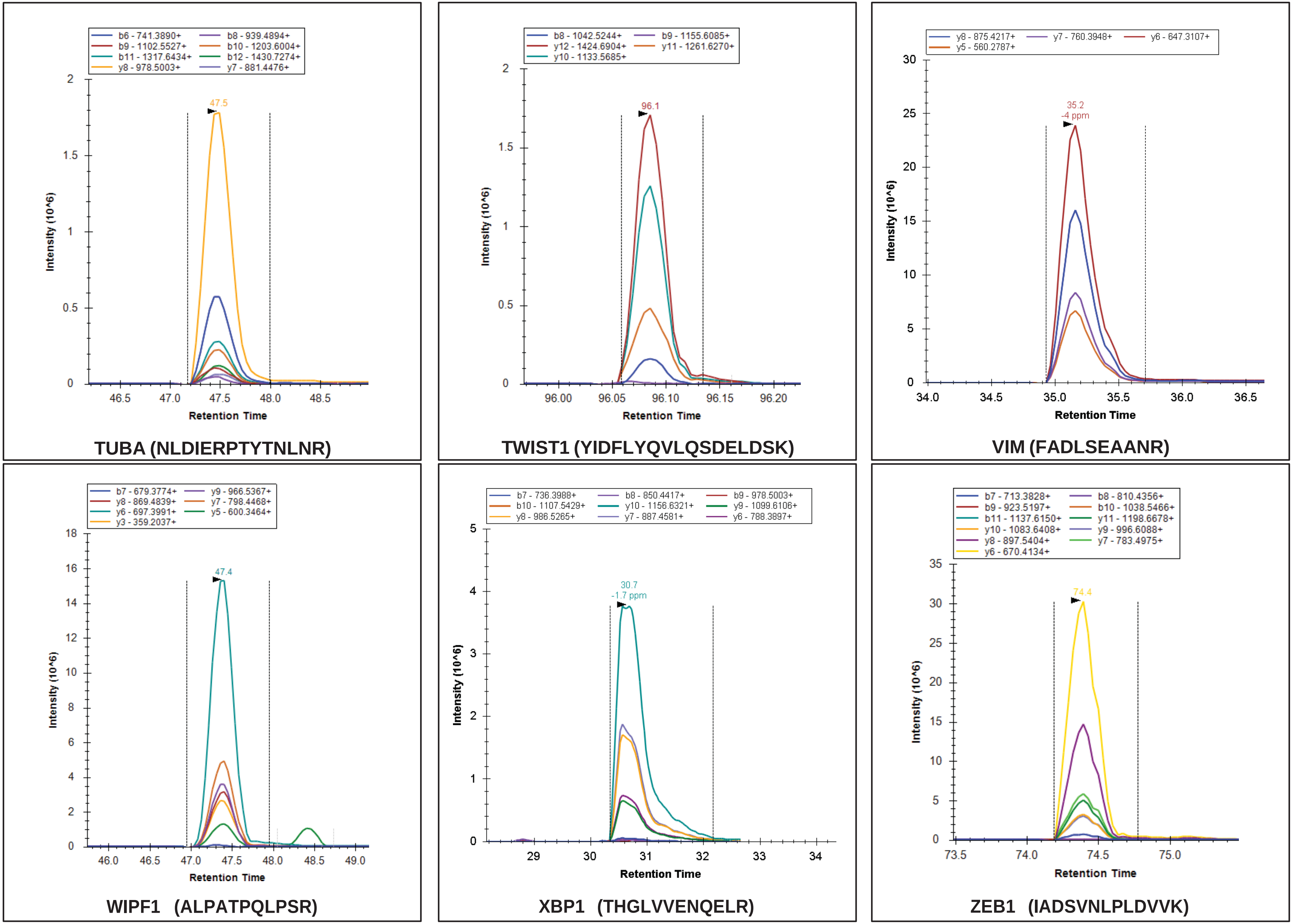
b6 - 741.3890+
b8 - 939.4894+
b9 - 1102.5527+
b10 - 1203.6004+
b11 - 1317.6434+
b12 - 1430.7274+
y8 - 978.5003+
y7 - 881.4476+
Intensity (10^6)
Retention Time
TUBA (NLDIERPTYTNLNR)
b8 - 1042.5244+
b9 - 1155.6085+
y12 - 1424.6904+
y11 - 1261.6270+
y10 - 1133.5685+
Intensity (10^6)
Retention Time
TWIST1 (YIDFLYQVLQSDELDSK)
y8 - 875.4217+
y7 - 760.3948+
y6 - 647.3107+
y5 - 560.2787+
35.2
-4 ppm
Intensity (10^6)
Retention Time
VIM (FADLSEAANR)
b7 - 679.3774+
y9 - 966.5367+
y8 - 869.4839+
y7 - 798.4468+
y6 - 697.3991+
y5 - 600.3464+
y3 - 359.2037+
Intensity (10^6)
Retention Time
WIPF1 (ALPATPQLPSR)
b7 - 736.3988+
b8 - 850.4417+
b9 - 978.5003+
b10 - 1107.5429+
y10 - 1156.6321+
y9 - 1099.6106+
y8 - 986.5265+
y7 - 887.4581+
y6 - 788.3897+
30.7
-1.7 ppm
Intensity (10^6)
Retention Time
XBP1 (THGLVVENQELR)
b7 - 713.3828+
b8 - 810.4356+
b9 - 923.5197+
b10 - 1038.5466+
b11 - 1137.6150+
y11 - 1198.6678+
y10 - 1083.6408+
y9 - 996.6088+
y8 - 897.5404+
y7 - 783.4975+
y6 - 670.4134+
Intensity (10^6)
Retention Time
ZEB1 (IADSVNLPLDVVK)

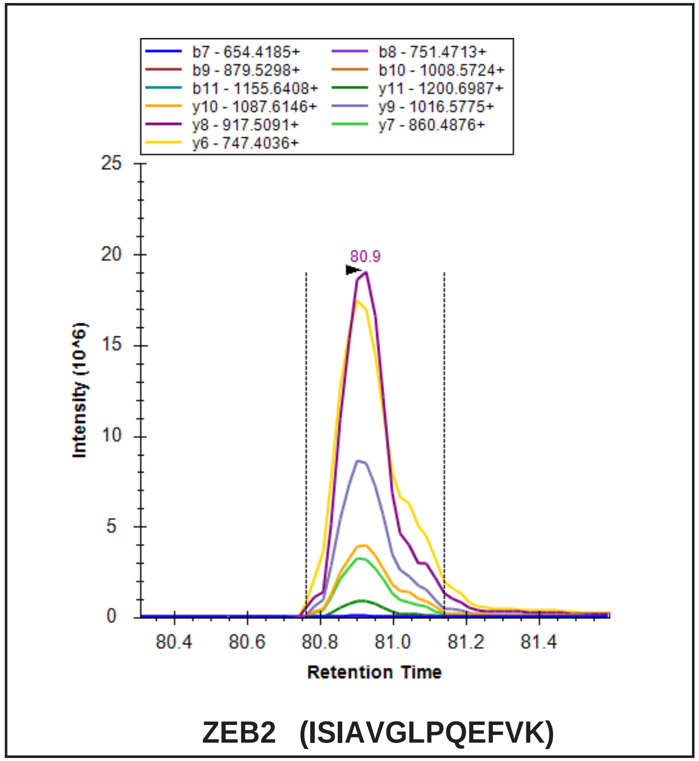
b7 - 654.4185+
b8 - 751.4713+
b9 - 879.5298+
b10 - 1008.5724+
b11 - 1155.6408+
y11 - 1200.6987+
y10 - 1087.6146+
y9 - 1016.5775+
y8 - 917.5091+
y7 - 860.4876+
y6 - 747.4036+
80.9
Intensity (10^6)
25
20
15
10
5
0
80.4
80.6
80.8
81.0
81.2
81.4
Retention Time
ZEB2 (ISIAVGLPQEFVK)

Supplementary Fig. 3 Calibration curve for 31 peptides precursors selected for monitoring protein expression across cell lines

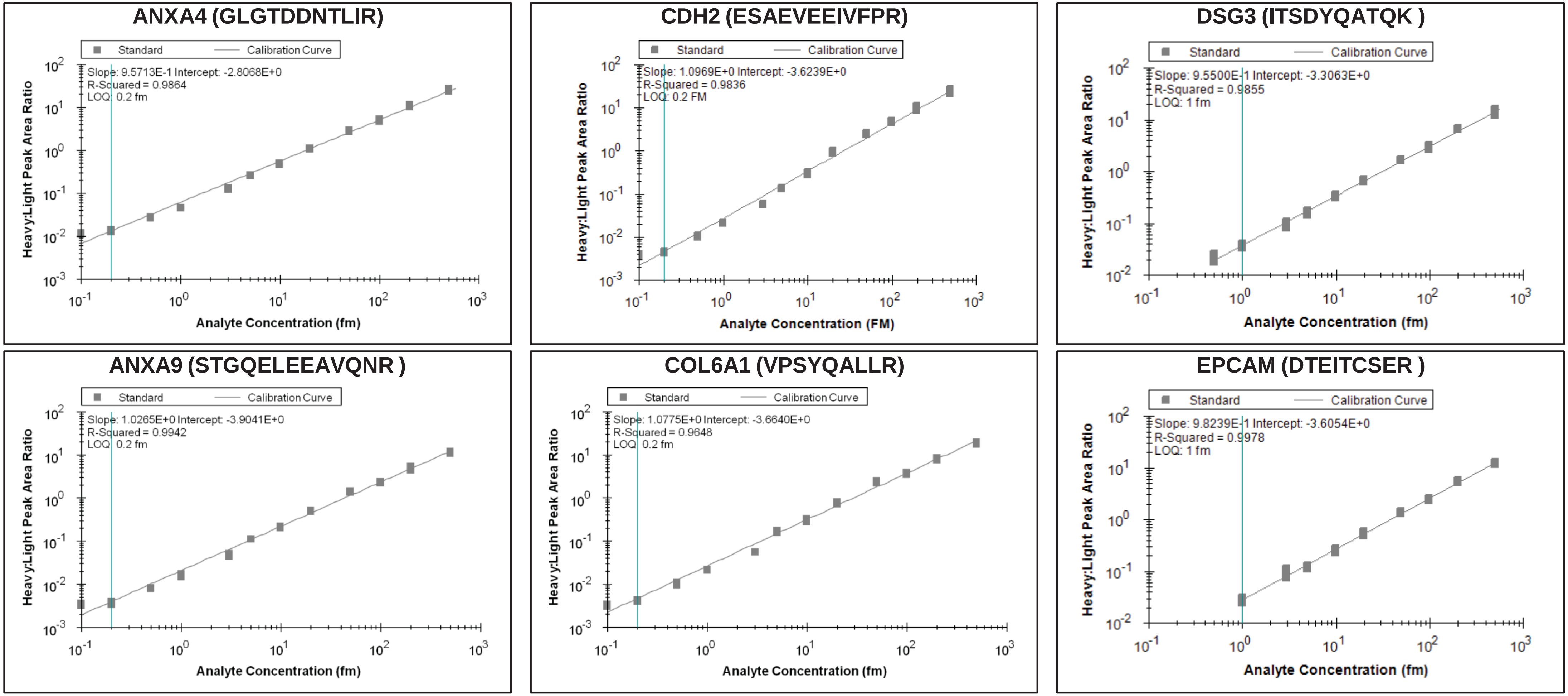

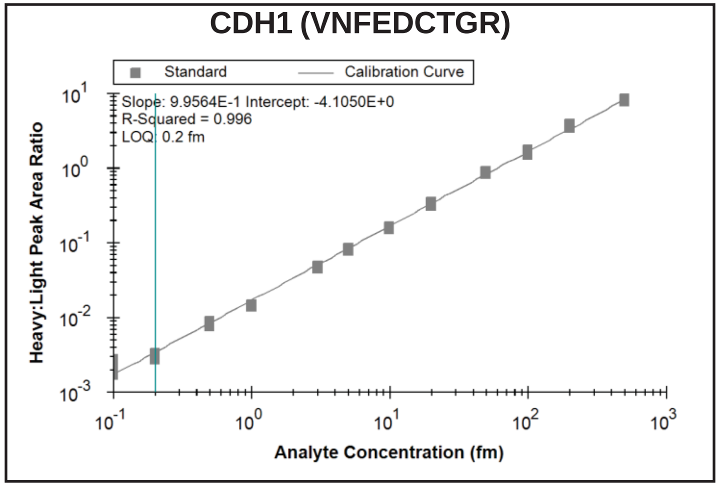
CDH1 (VNFEDCTGR)
Standard
Calibration Curve
Slope: 9.9564E-1 Intercept: -4.1050E+0
R-Squared = 0.996
LOQ: 0.2 fm
Heavy:Light Peak Area Ratio
Analyte Concentration (fm)

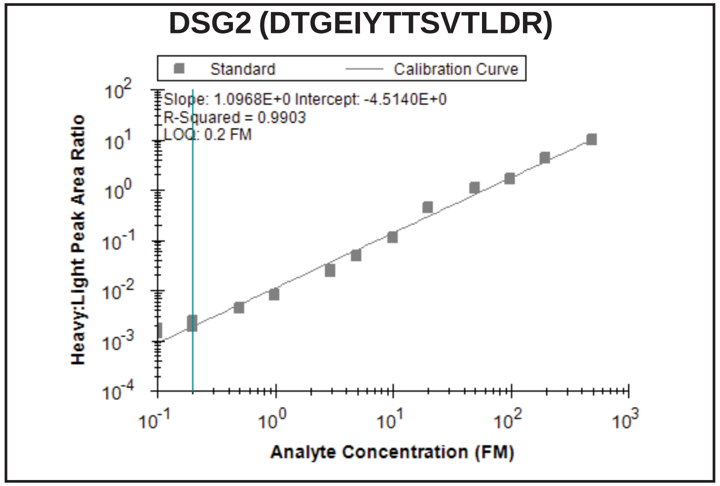
DSG2 (DTGEIYTTSVTLDR)
Standard
Calibration Curve
Slope: 1.0968E+0 Intercept: -4.5140E+0
R-Squared = 0.9903
LOQ: 0.2 FM
Heavy:Light Peak Area Ratio
Analyte Concentration (FM)

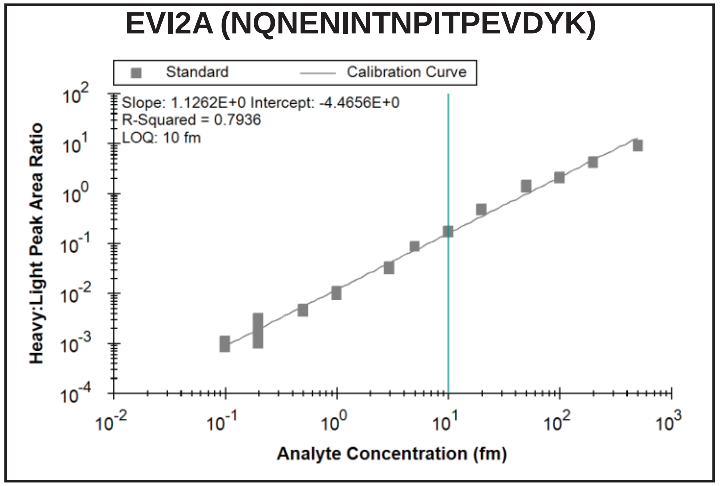
EVI2A (NQNENINTNPITPEVDYK)
Standard
Calibration Curve
Slope: 1.1262E+0 Intercept: -4.4656E+0
R-Squared = 0.7936
LOQ: 10 fm
Heavy:Light Peak Area Ratio
Analyte Concentration (fm)

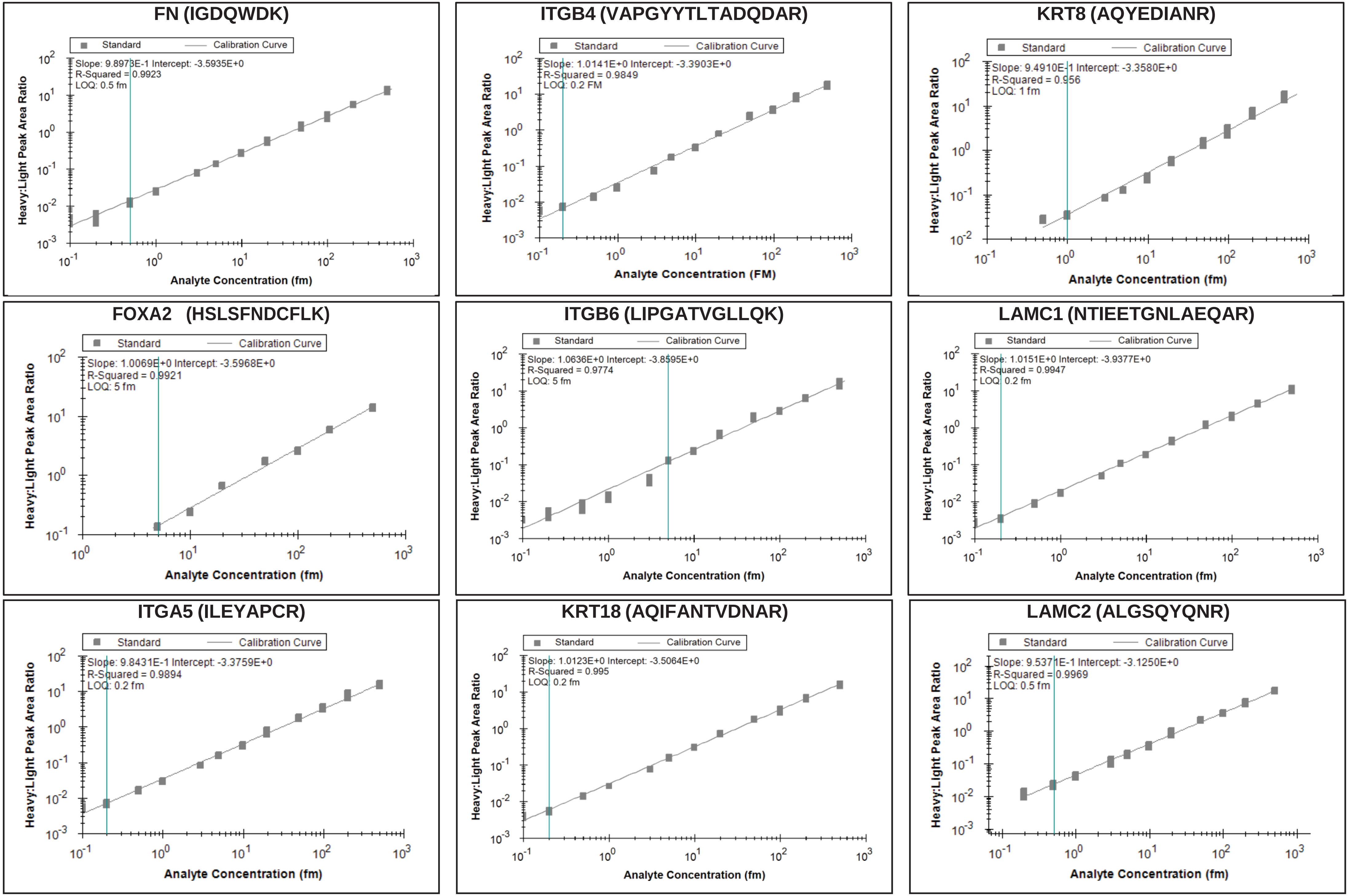
FN (IGDQWDK)
Standard
Calibration Curve
Slope: 9.8973E-1 Intercept: -3.5935E+0
R-Squared = 0.9923
LOQ: 0.5 fm
Heavy:Light Peak Area Ratio
Analyte Concentration (fm)
ITGB4 (VAPGYYTLTADQDAR)
Standard
Calibration Curve
Slope: 1.0141E+0 Intercept: -3.3903E+0
R-Squared = 0.9849
LOQ: 0.2 FM
Heavy:Light Peak Area Ratio
Analyte Concentration (FM)
KRT8 (AQYEDIANR)
Standard
Calibration Curve
Slope: 9.4910E-1 Intercept: -3.3580E+0
R-Squared = 0.956
LOQ: 1 fm
Heavy:Light Peak Area Ratio
Analyte Concentration (fm)
FOXA2 (HSLSFNDCFLK)
Standard
Calibration Curve
Slope: 1.0069E+0 Intercept: -3.5968E+0
R-Squared = 0.9921
LOQ: 5 fm
Heavy:Light Peak Area Ratio
Analyte Concentration (fm)
ITGB6 (LIPGATVGLLQK)
Standard
Calibration Curve
Slope: 1.0636E+0 Intercept: -3.8595E+0
R-Squared = 0.9774
LOQ: 5 fm
Heavy:Light Peak Area Ratio
Analyte Concentration (fm)
LAMC1 (NTIEETGNLAEQAR)
Standard
Calibration Curve
Slope: 1.0151E+0 Intercept: -3.9377E+0
R-Squared = 0.9947
LOQ: 0.2 fm
Heavy:Light Peak Area Ratio
Analyte Concentration (fm)
ITGA5 (ILEYAPCR)
Standard
Calibration Curve
Slope: 9.8431E-1 Intercept: -3.3759E+0
R-Squared = 0.9894
LOQ: 0.2 fm
Heavy:Light Peak Area Ratio
Analyte Concentration (fm)
KRT18 (AQIFANTVDNAR)
Standard
Calibration Curve
Slope: 1.0123E+0 Intercept: -3.5064E+0
R-Squared = 0.995
LOQ: 0.2 fm
Heavy:Light Peak Area Ratio
Analyte Concentration (fm)
LAMC2 (ALGSQYQNR)
Standard
Calibration Curve
Slope: 9.5371E-1 Intercept: -3.1250E+0
R-Squared = 0.9969
LOQ: 0.5 fm
Heavy:Light Peak Area Ratio
Analyte Concentration (fm)

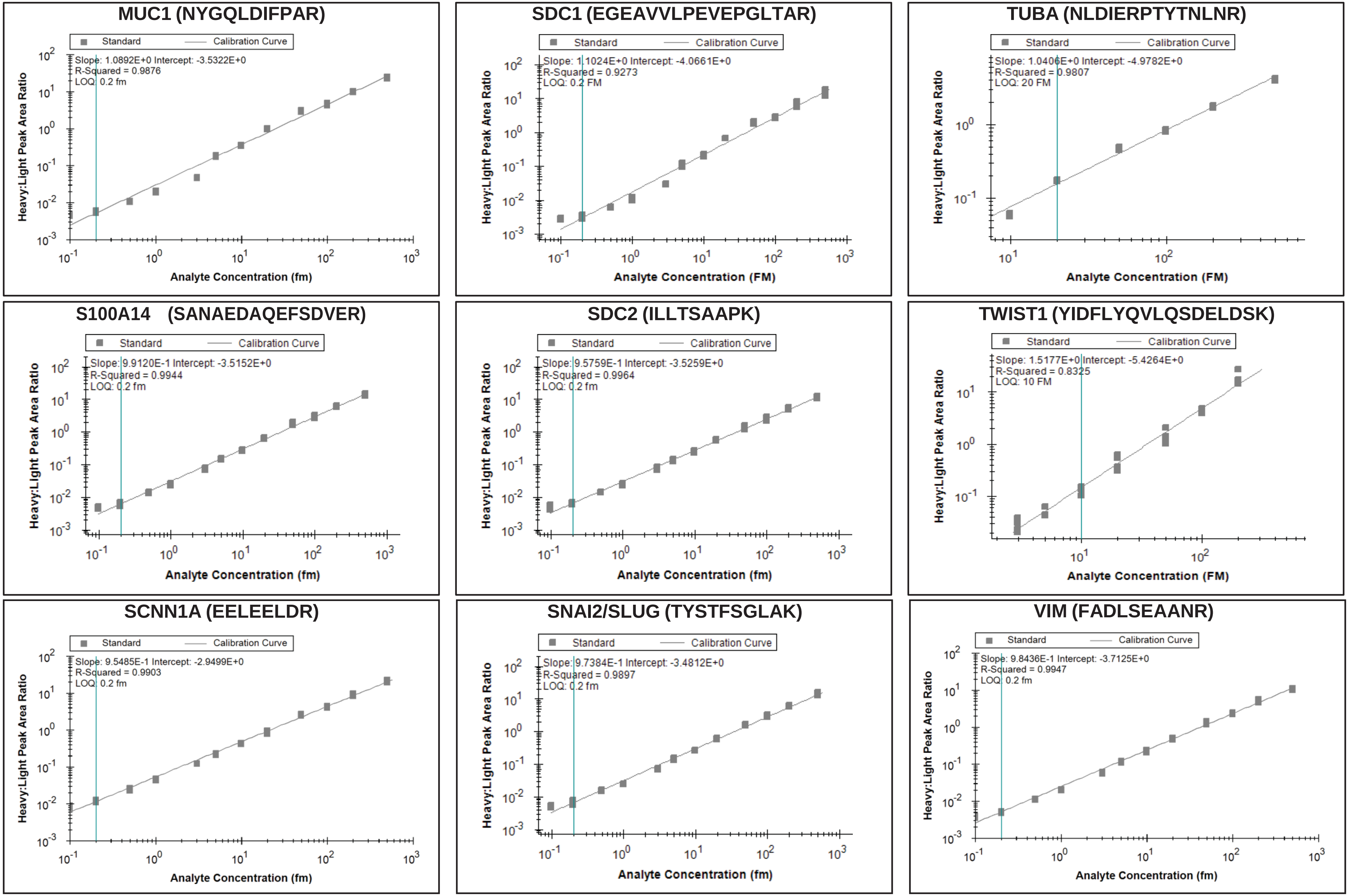

MUC1 (NYGQLDIFPAR)
Standard
Calibration Curve
Slope: 1.0892E+0 Intercept: -3.5322E+0
R-Squared = 0.9876
LOQ: 0.2 fm
Heavy:Light Peak Area Ratio
Analyte Concentration (fm)
SDC1 (EGEAVVLPEVEPGLTAR)
Standard
Calibration Curve
Slope: 1.1024E+0 Intercept: -4.0661E+0
R-Squared = 0.9273
LOQ: 0.2 FM
Heavy:Light Peak Area Ratio
Analyte Concentration (FM)
TUBA (NLDIERPTYTNLNR)
Standard
Calibration Curve
Slope: 1.0406E+0 Intercept: -4.9782E+0
R-Squared = 0.9807
LOQ: 20 FM
Heavy:Light Peak Area Ratio
Analyte Concentration (FM)
S100A14 (SANAEDAQEFSDVER)
Standard
Calibration Curve
Slope: 9.9120E-1 Intercept: -3.5152E+0
R-Squared = 0.9944
LOQ: 0.2 fm
Heavy:Light Peak Area Ratio
Analyte Concentration (fm)
SDC2 (ILLTSAAPK)
Standard
Calibration Curve
Slope: 9.5759E-1 Intercept: -3.5259E+0
R-Squared = 0.9964
LOQ: 0.2 fm
Heavy:Light Peak Area Ratio
Analyte Concentration (fm)
TWIST1 (YIDFLYQVLQSDELDSK)
Standard
Calibration Curve
Slope: 1.5177E+0 Intercept: -5.4264E+0
R-Squared = 0.8325
LOQ: 10 FM
Heavy:Light Peak Area Ratio
Analyte Concentration (FM)
SCNN1A (EELEELDR)
Standard
Calibration Curve
Slope: 9.5485E-1 Intercept: -2.9499E+0
R-Squared = 0.9903
LOQ: 0.2 fm
Heavy:Light Peak Area Ratio
Analyte Concentration (fm)
SNAI2/SLUG (TYSTFSGLAK)
Standard
Calibration Curve
Slope: 9.7384E-1 Intercept: -3.4812E+0
R-Squared = 0.9897
LOQ: 0.2 fm
Heavy:Light Peak Area Ratio
Analyte Concentration (fm)
VIM (FADLSEAANR)
Standard
Calibration Curve
Slope: 9.8436E-1 Intercept: -3.7125E+0
R-Squared = 0.9947
LOQ: 0.2 fm
Heavy:Light Peak Area Ratio
Analyte Concentration (fm)

ZEB1 (IADSVNLPLDVVK)

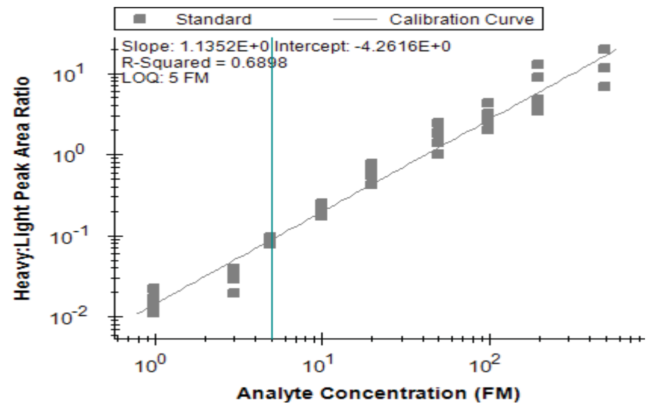

ZEB2 (ISIAVGLPQEFVK)

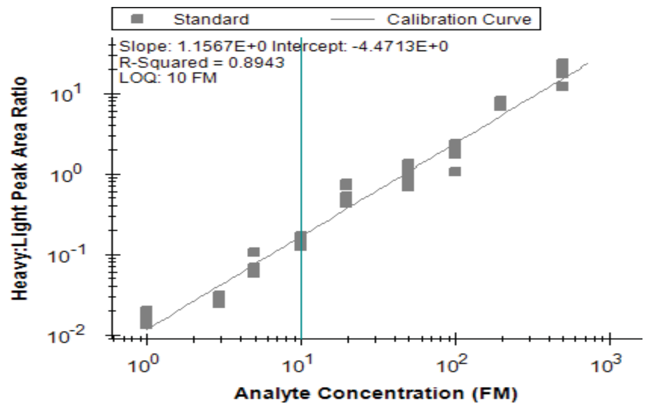

WIPF1 (ALPATPQLPSR)

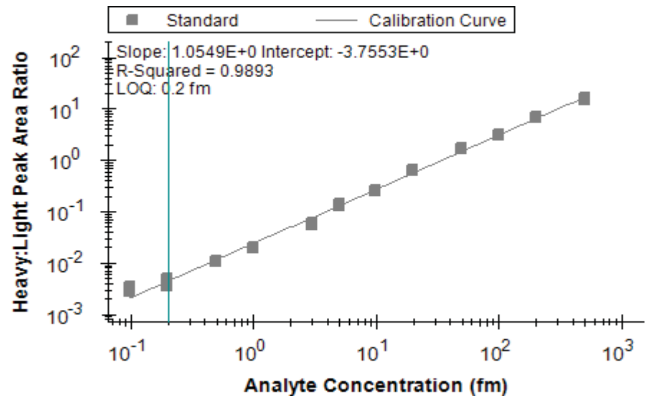

XBP1 (THGLVVENQELR)

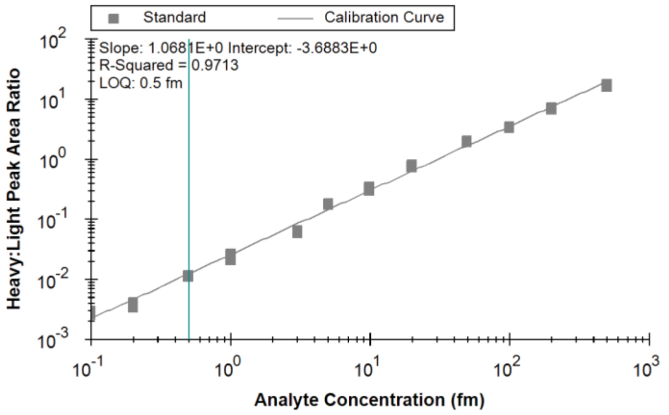

Supplementary Fig. 4 Co-relation plots for mRNA-based gene expression and protein expression for values for depicted cell lines based on transcriptomics and reversed phase protein array (RPPA) proteomics data from CCLE database

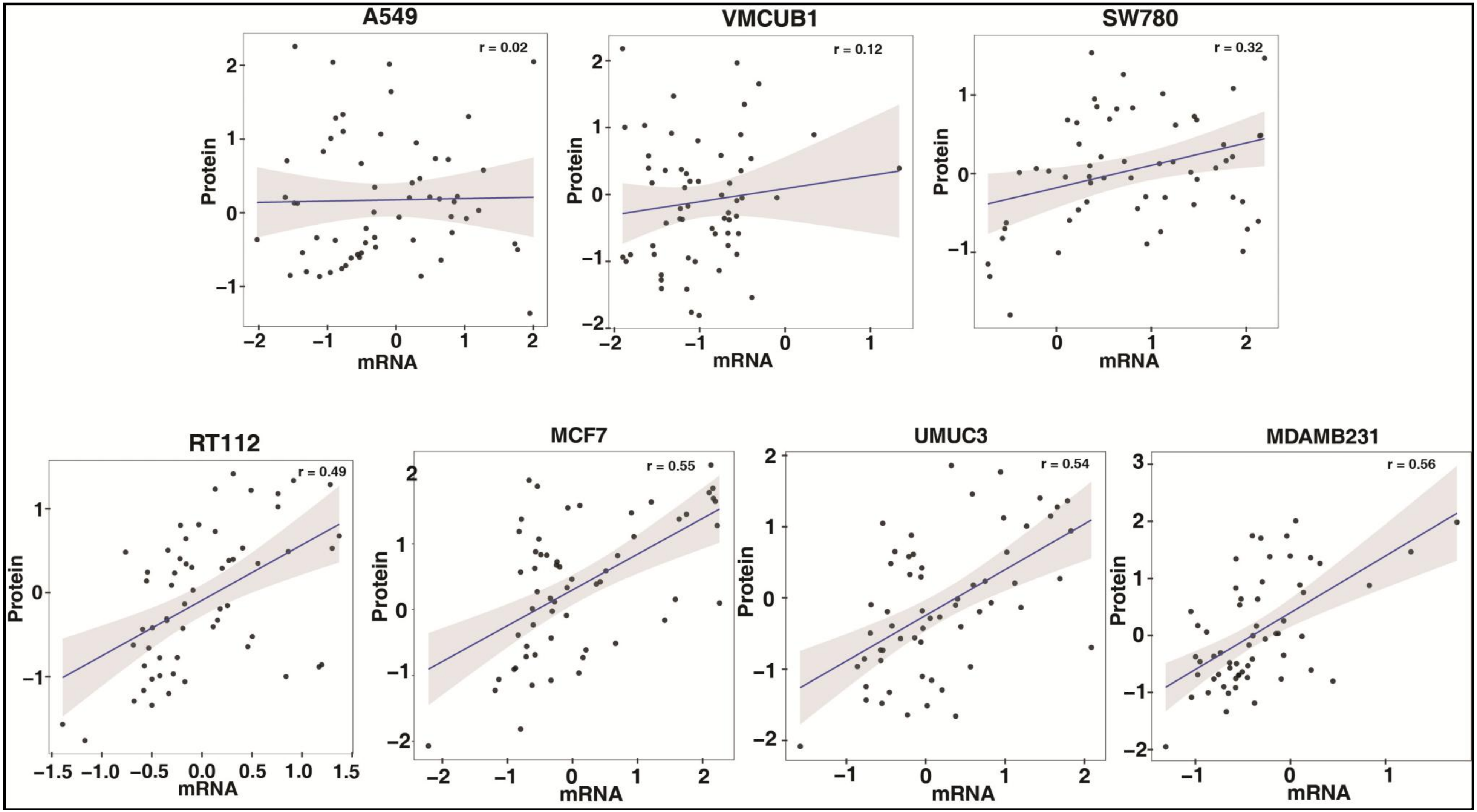

Supplementary Method 1: The overview of all the cell lines used in the study along with the tissue of origin and culture method

Cell line	Tissue of origin	Culture Conditions
VMUC1	BLADDER	Dulbecco's Modified Eagle Medium (DMEM) with fetal bovine serum (FBS) to a final concentration of 10% (adapted inhouse culture method)
RT112	BLADDER	Dulbecco's Modified Eagle Medium (DMEM) with fetal bovine serum (FBS) to a final concentration of 10% (adapted inhouse culture method)
SW780	BLADDER	Dulbecco's Modified Eagle Medium (DMEM) with fetal bovine serum (FBS) to a final concentration of 10% (adapted inhouse culture method)
J82	BLADDER	Dulbecco's Modified Eagle Medium (DMEM) with fetal bovine serum (FBS) to a final concentration of 10% (adapted inhouse culture method)
UMUC3	BLADDER	Dulbecco's Modified Eagle Medium (DMEM) with fetal bovine serum (FBS) to a final concentration of 10% (adapted inhouse culture method)
HeLa	CERVIX	Dulbecco's Modified Eagle Medium (DMEM) low glucose with fetal bovine serum (FBS) to a final concentration of 5% (adapted inhouse culture method)
SiHa	CERVIX	Eagle's Minimum Essential Medium with fetal bovine serum (FBS) to a final concentration of 10% (ATCC culture method)
CaL27	ORAL	Dulbecco's Modified Eagle Medium (DMEM) with fetal bovine serum (FBS) to a final concentration of 10% (adapted inhouse culture method)
FaDu	ORAL	Eagle's Minimum Essential Medium with fetal bovine serum (FBS) to a final concentration of 10% (ATCC culture method)
TE1	ESOPHAGEAL	Dulbecco's Modified Eagle Medium (DMEM) with fetal bovine serum (FBS) to a final concentration of 10% (adapted inhouse culture method)
H358	LUNG	RPMI-1640 Mediumwith 10% fetal bovine serum (FBS) to a final concentration of 10% (ATCC culture method)
A549	LUNG	Dulbecco's Modified Eagle Medium F-12 (DMEM F-12) with fetal bovine serum (FBS) to a final concentration of 10% (adapted inhouse culture method)
MCF7	BREAST	Dulbecco's Modified Eagle Medium (DMEM) low glucose with fetal bovine serum (FBS) to a final concentration of 5% (adapted inhouse culture method)
MDAMB231	BREAST	Dulbecco's Modified Eagle Medium (DMEM) with fetal bovine serum (FBS) to a final concentration of 10% (adapted inhouse culture method)
AGS	GASTRIC	F-12K Medium with with fetal bovine serum (FBS) to a final concentration of 10% (ATCC culture method)
G415	GALL BLADDER	Dulbecco's Modified Eagle Medium (DMEM) with fetal bovine serum (FBS) to a final concentration of 10% (adapted inhouse culture method)
OCUG1	GALL BLADDER	Dulbecco's Modified Eagle Medium (DMEM) with fetal bovine serum (FBS) to a final concentration of 10% (adapted inhouse culture method)
NOZ	GALL BLADDER	Dulbecco's Modified Eagle Medium (DMEM) high glucose and low glucose (1:1) with fetal bovine serum(FBS) to a final concentration of 10% (adapted inhouse culture method)
